# Metagenomic Analysis of the Sponge *Discodermia* Reveals the Production of the Cyanobacterial Natural Product Kasumigamide by ‘Entotheonella’

**DOI:** 10.1371/journal.pone.0164468

**Published:** 2016-10-12

**Authors:** Yu Nakashima, Yoko Egami, Miki Kimura, Toshiyuki Wakimoto, Ikuro Abe

**Affiliations:** 1 Graduate School of Pharmaceutical Sciences, The University of Tokyo, Bunkyo-ku, Tokyo, Japan; 2 Graduate School of Pharmaceutical Sciences, Hokkaido University, Kita-ku, Sapporo, Japan; University of New South Wales, AUSTRALIA

## Abstract

Sponge metagenomes are a useful platform to mine cryptic biosynthetic gene clusters responsible for production of natural products involved in the sponge-microbe association. Since numerous sponge-derived bioactive metabolites are biosynthesized by the symbiotic bacteria, this strategy may concurrently reveal sponge-symbiont produced compounds. Accordingly, a metagenomic analysis of the Japanese marine sponge *Discodermia calyx* has resulted in the identification of a hybrid type I polyketide synthase-nonribosomal peptide synthetase gene (*kas*). Bioinformatic analysis of the gene product suggested its involvement in the biosynthesis of kasumigamide, a tetrapeptide originally isolated from freshwater free-living cyanobacterium *Microcystis aeruginosa* NIES-87. Subsequent investigation of the sponge metabolic profile revealed the presence of kasumigamide in the sponge extract. The kasumigamide producing bacterium was identified as an ‘Entotheonella’ sp. Moreover, an *in silico* analysis of *kas* gene homologs uncovered the presence of *kas* family genes in two additional bacteria from different phyla. The production of kasumigamide by distantly related multiple bacterial strains implicates horizontal gene transfer and raises the potential for a wider distribution across other bacterial groups.

## Introduction

**Fig 1:**
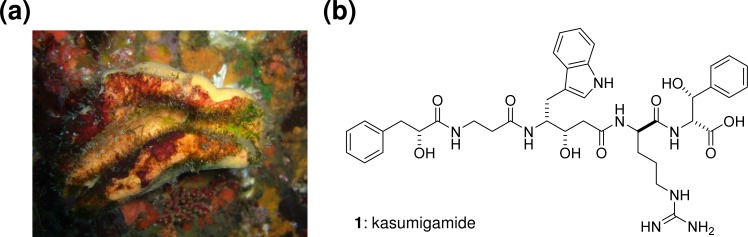
Kasumigamide from the marine sponge *Discodermia calyx*. **(a)** The marine sponge *Discodermia calyx*. **(b)** Structure of kasumigamide.

Metagenome mining strategies [**1**] are applicable for the discovery of unknown compounds, particularly polyketides and nonribosomal peptides, from uncultured bacteria. Based on the colinearity rule of domain and module organization of modular polyketide synthase (PKS) and nonribosomal peptide synthetases (NRPS) [**2–3**], it is possible to predict the chemical structures of products derived from orphan gene clusters comprising PKS and/or NRPS genes. As many polyketides and nonribosomal peptides have been isolated from marine sponges, and there is strong evidence that those natural products are actually produced by their microbial symbionts, sponge metagenomes have become a useful source for identifying the real producers of those natural products. Among the many different sponge symbionts, e.g., Proteobacteria, Actinobacteria, Acidobacteria, Cyanobacteria [**4**], the ‘Enthotheonella’ group belonging to the candidate phylum ‘Tectomicrobia’ has recently been described as a highly prolific bacterial symbiont phylotype capable of producing structurally complex bioactive molecules [**5–6**]. This as-yet-uncultured group of bacteria has been frequently detected in several marine sponges. In the marine sponges *Theonella swinhoei* Y and *T*. *swinhoei* WA, ‘Entotheonella’ was identified as the true producer of a number of natural products [**7–8**]. The Japanese marine sponge *Discodermia calyx* (**Fig 1A**) [**9**] is also known as a rich source of bioactive compounds, such as calyculins and calyxamides [**10–12**]. Through extensive screening of the metagenomic library of *D*. *calyx*, we recently identified the calyculin biosynthetic gene cluster and linked this cluster to the symbiotic bacterium, ‘Entotheonella’ sp. by employing single cell analysis [**13**].

Furthermore, we employed degenerate PCR to target gene within the *D*. *calyx* metagenome that encode ketosynthase (KS) domains [**14–18**] and found a range of distinct KS domain sequences. Of these, several were associated with *trans*-acyl transferase (AT) type KS domains and characterized as components of the calyculin biosynthetic gene cluster. Other KS domains, however, could not be attributed to a previously characterized biosynthetic pathway. Therefore, to search for new biosynthetic gene clusters and their products, extensive characterization of the diverse KS domains present in the *D*. *calyx* metagenomic library is warranted. Here, we present such a study, targeting KS domains affiliated with the *cis*-AT KS group and subsequently characterizing the production of the cyanobacterial natural product kasumigamide by a sponge symbiont.

## Results

### Annotation and predicted product of *kas* gene cluster

**Fig 2:**
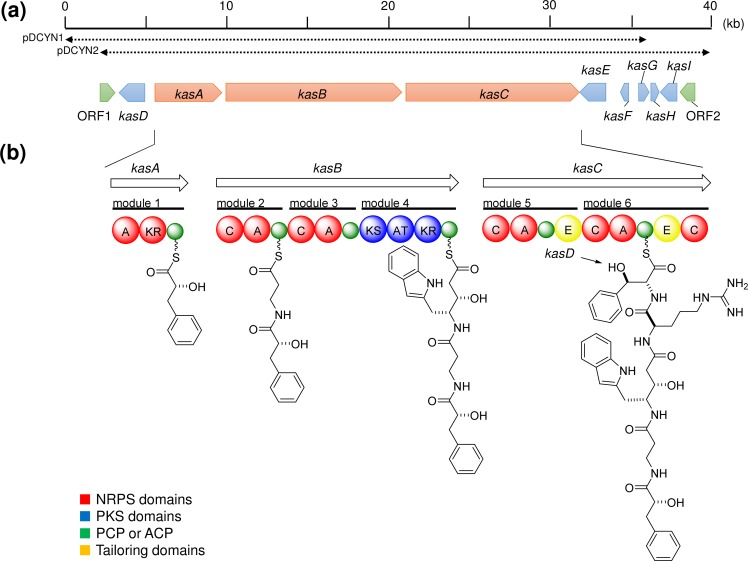
The biosynthetic gene cluster and proposed biosynthetic pathway to kasumigamide. **(a)** ORFs encoded in the putative kasumigamide biosynthetic gene cluster, *kasA-I*. Double-headed arrows show the location of pDCYN1-2. The ORFs related to PKS-NRPS are highlighted in red. Putative transposases are colored in green. **(b)** The domain organization and proposed biosynthetic pathway to kasumigamide.

Screening of the *D*. *calyx* fosmid library using specific primers for the *cis*-AT KS domain resulted in the identification of two clones pDCYN1 and pDCYN2. Sequencing of the fosmid clones revealed the existence of a hybrid PKS-NRPS biosynthetic gene cluster (*kasA-I*, ~37 kb, LC160290) (**Table 1**, **Fig 2**), composed of 9 open reading frames (ORFs), with *kasA-C* forming a PKS-NRPS core. KasA is comprised of an adenylation (A) domain (KasA-A1), a ketoreductase (KR) domain (KasA-KR), and a peptidyl carrier protein (PCP) domain. KasB consists of three modules (modules 2–4): the first two modules encode NRPSs, and the latter encodes a PKS. The two following NRPS modules (modules 5–6) are encoded in *kasC*. The substrates for the five A domains (KasA-A1, KasB-A1, A2, KasC-A1, and A2) were predicted from the NRPS codes [**19**] with the exception of KasA-A1, which was unmatched to known data, but annotated to the A domain recruiting phenyl pyruvic acid (PP) based on the close resemblance to AerA of aeruginosin biosynthetic gene cluster (**S2 Table**) [**20**]. Collectively, the chemical structure of the PKS and NRPS hybrid compound was predicted to be kasumigamide, which had previously been isolated from the freshwater cyanobacterium *Microcystis aeruginosa* NIES-87 (**Fig 1B**) [**21**]. However there have been no reports of the isolation of this compound from marine sponges.

**Table 1:** Putative ORFs of kasumigamide NRPS-PKS gene cluster derived from “Entotheonella” sp.

ORFs	Protein size (aa)	Proposed function	Sequence similarity, protein (origin)	Identity/Similarity (%)	Accession number
ORF1	305	Transposase	Transposase (*Rhodopirellula europaea*)	27/47	WP_008672175
*kasD*	529	Hydroxylase	CmlA (*Streptomyces Venezuelae*)	39/57	WP_015032127
*kasA*	1381	NRPS (A, KR, PCP)			
*kasB*	3694	PKS/NRPS (C, A, PCP, C, A, PCP, KS, AT, KR, ACP)			
*kasC*	3560	NRPS (C, A, PCP, E, C, A, PCP, E, C)			
*kasE*	540	Transporter	Cyclic peptide transporter (*Methylobacter tundripaludum*)	38/59	WP_006892918
*kasF*	168	Transcriptional regulator	Transcriptional regulator (*Desulfurivibrio alkaliphilus*)	36/54	WP_013162517
*kasG*	215	Methyltransferase	Methyltransferase (*Rhodococcus hoagie*)	38/55	WP_022594278
*kasH*	173	Chorismate synthase	Chorismate synthase (*Kribbella catacumbae*)	39/50	WP_020385871
*kasI*	341	Hypothetical protein	Hypothetical protein ETSY1_01160 (Candidatus “Entotheonella” sp. TSY1)	92/94	ETX03129
ORF2	305	Transposase	Transposase (*Rhodopirellula europaea*)	27/47	WP_008672175

### Isolation of kasumigamide from marine sponge *D*. *calyx*

In order to search for kasumigamide and related compounds, the MeOH extract of the frozen sponge *D*. *calyx* was fractionated by column chromatography, based on LC-MS monitoring, to yield the putative *kas* gene product **1**.

The molecular formula of **1** was established by ESI-TOFMS to be C_40_H_50_N_8_O_9_ [*m/z* 809.3801 (M+Na)^+^ for C_40_H_50_N_8_NaO_9_], which is consistent with that of kasumigamide (**S1 Fig**). Furthermore, 1D and 2D NMR data of **1**, including ^1^H-^1^H COSY, HMQC, HMBC, and NOESY spectra in DMSO-*d*_6_+TFA, suggested the presence of β-Ala and Arg (**S3 Table**) (**S4 Table**) (**S2 Fig**) (**S3 Fig**) (**S4 Fig**) (**S5 Fig**) (**S6 Fig**) [**21**]. The four aromatic protons (δ_H_ = 6.91, 7.00, 7.28, and 7.47) and an exchangeable proton (δ_H_ = 10.74) coupled to another aromatic proton (δ_H_ = 7.00) were consistent with an indole ring, supported by the UV absorption maxima at 280 nm. In addition, the other ten aromatic protons at δ_H_ = 7.10–7.23 overlapped, which suggested the presence of two mono-substituted benzene rings. The HMBC correlation between aromatic protons (δ_H_ = 7.23) and an oxymethine carbon (δ_C_ 75.9) indicated the presence of 3-phenylserine. In contrast, other aromatic protons (δ_H_ = 7.16) showed HMBC correlation with a methylene carbon (δ_C_ 41.2), which further correlated with an oxymethine proton at δ_H_ = 4.00, supporting the presence of phenyl lactic acid. The amino acid sequence of **1** was confirmed by NOESY and HMBC correlations. The ODS HPLC analysis of L-FDAA derivatives of the hydrolysate of **1** revealed the presence D-Arg and D-*erythro*-3-phenylserine (D-*erythro*-PS). Therefore, the gross structure was concluded to be **1**, which is coincident with the predicted product of the PKS and NRPS hybrid gene cluster, *kasA-I*.

### Kasumigamide producer in the marine sponge

**Fig 3:**
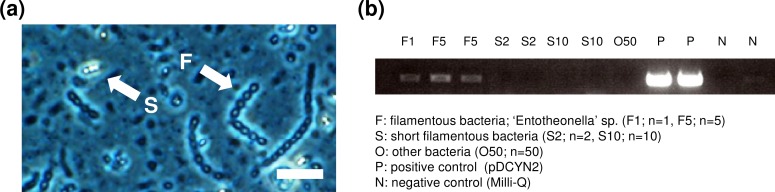
Symbiont bacteria bearing *kas* genes. **(a)** Phase contrast image of *D*. *calyx* homogenate. A filamentous bacterium ‘Entotheonella’ sp. is designated as “F”. A small filamentous bacterium with bright color is designated as “S”. Scale bars was 20 μm. **(b)** PCR analysis of dissected cells with the *kas*-specific primers pair, DCKS10F/DCKS10R (**S1 Table**), using dissected cells (“F” or “S”) as templates.

To identify the true producer of kasumigamide in *D*. *calyx*, we used the laser microdissection (LMD) method to isolate the symbiont cells for PCR analysis. As the candidates, two types of cells designated as “F” and “S” filamentous morphologies (**Fig 3A**) were isolated from the sponge material. Of these two, only cells with the “F” morphology (**Fig 3A**) were returned positive when the genomes were amplified using the *kas* specific primer pair (**Fig 3B**). This filamentous bacterium was previously reported to be ‘Entotheonella’ sp. as the producer of calyculins in *D*. *calyx* (**Fig 3A**) [**13**].

### Kasumigamide biosynthetic gene cluster in cyanobacterium

**Fig 4:**
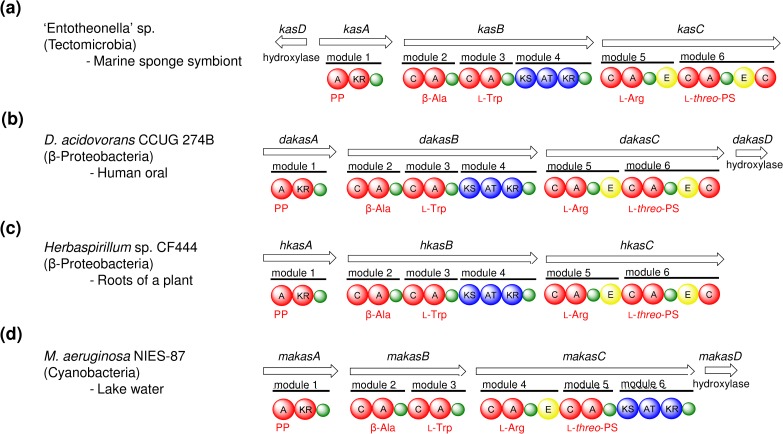
Comparative analysis of domain organizations of the putative kasumigamide biosynthetic gene clusters. Each gene cluster derived from **(a)** *D*. *acidovorans* CCUG 274B **(b)** *Herbaspirillum* sp. CF444 **(c)** ‘Entotheonella’ sp. **(d)** *M*. *aeruginosa* NIES-87.

Kasumigamide was first identified in the free-living cyanobacterium *M*. *aeruginosa* NIES-87 [**21**]; however, its biosynthetic pathway in this organism was unknown. To compare the corresponding gene clusters between the two different bacterial species, we set out to identify a kasumigamide biosynthetic gene cluster in *M*. *aeruginosa* NIES-87. First, the ability of *M*. *aeruginosa* NIES-87 to produce kasumigamide was confirmed by LC-MS analysis (**S7 Fig**). One of the *M*. *aeruginosa* metabolites showed the same retention time and molecular mass as those of kasumigamide isolated from *D*. *calyx*. These results corroborated the previous report of kasumigamide production in *M*. *aeruginosa* NIES-87 [**21**]. The genome was then sequenced and assembled (see Materials and Methods). A homology-based search revealed the existence of the PKS-NRPS biosynthetic gene cluster, named *makasA-D* (**S5 Table**, LC160291) (**S8C Fig**). The *makasA-C* genes encode five NRPS modules and one PKS module (**Fig 4D**).

### Substrate specificity of the adenylation domains

**Fig 5:**
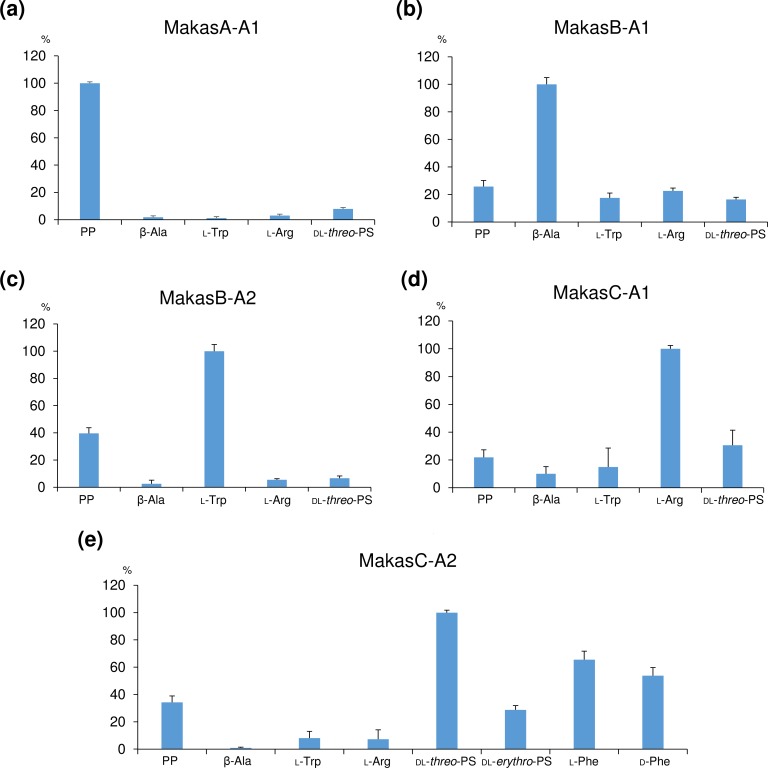
Substrate selectivity of A domains. The relative adenylation activity was estimated by the malachite green phosphate assay. Error bars represent SEM (n = 3).

In order to confirm the predicted substrates for the A domains in MakasA-C, an *in vitro* analysis was performed using the Biomol Green assay strategy [**22–23**]. The His6-tagged recombinant proteins of the five A domains, MakasA-A1, MakasB-A1, MakasB-A2, MakasC-A1, and MakasC-A2, were produced in *Escherichia coli* and purified by nickel-nitrilotriacetic acid (Ni-NTA) affinity chromatography (**S9 Fig**). Maltose binding protein (MBP) was used to improve the solubility of MakasB2 and MakasC1. In the case of MakasB-A1 and MakasC-A2, the C and PCP domains were coexpressed. MakasA-A1, MakasB-A1, MakasB-A2, and MakasC-A1 exhibited catalytic activities selective for PP, β-Ala, L-Trp, and L-Arg, respectively (**Fig 5**). In addition, MakasC-A2 showed higher selectivity to DL-*threo*-3-phenylserine (DL-*threo*-PS) than L-Phe, in accord to the substrate binding prediction (**S2 Table**). However, we have so far not succeeded in the heterologous expression of each A domain of KasA-C as well as the entire gene cluster, *kasA-I* using *E*. *coli*, *Streptomyces lividans*, or *Streptomyces avermitilis* as a host.

### Kasumigamide biosynthetic gene cluster in phylogenetically diverse bacteria

The above studies revealed that kasumigamide is produced by two phylogenetically distant bacterial species, ‘Entotheonella’ sp. and *M*. *aeruginosa* NIES-87. This observation suggests a horizontal transfer of kasumigamide biosynthetic gene cluster can occur between different bacterial species. Therefore, we searched for additional *kas* biosynthetic gene clusters using BLASTP, with KasA-D as a query. As a result, two candidate clusters were found in the β-proteobacteria, *Delftia acidovorans* CCUG 274B (WP_016445283-WP_016445295) and *Herbaspirillum* sp. CF444 (EJL94052-EJL94061), and the putative kasumigamide biosynthetic gene clusters were annotated and named *dakasA-J* and *hkasA-H*, respectively (**S6 Table**) (**S7 Table**) (**S8A and S8B Fig**) [**24**]. The GC contents of *kasA-I*, *makasA-D*, *dakasA-J*, and *hkasA-H* are 57.5%, 52.9%, 70.6%, and 64.7% respectively. The domain organizations of PKS-NRPS modules of *dakasA-J* and *hkasA-H* were identical to those of *kasA-C* (**Fig 4A–4C**). The A domain binding sites exhibit 77~100% identity to the corresponding domains of KasA-C and/or MakasA-C (**S2 Table**). Finally, phylogenetic analysis of the KS domains (**S10 Fig**) shows their close relationships to each other despite that they are originated from phylogenetically distinct bacteria (**S11 Fig**).

## Discussion

The strategy for isolating kasumigamide from *D*. *calyx* exemplifies the effectiveness of the metagenome mining approach in identifying cryptic biosynthetic gene clusters as well as their products. The involvement of the *kas* cluster in kasumigamide biosynthesis was predicted based on its domain organization (**Fig 2**). Whereas the NRPS code of KasA-A1 did not provide any information about its natural substrate (**S2 Table**), KasA-KR resembles the KR domain of AerA (~41% identity), which reportedly reduces PP to generate D-phenyl lactic acid [**20**]. KasD was annotated as a β-hydroxylase, according to its similarity to CmlA [**25**] (~39% identity). CmlA catalyzes β-hydroxylation of L-p-aminophenylalanine (L-PAPA) to L-p-aminophenylserine (L-PAPS), and the metal ion binding site of CmlA are also well conserved in KasD (**S12 Fig**). Since L-Phe is structurally similar to L-PAPA, KasD was proposed to catalyze the β-hydroxylation of L-Phe, to generate L-*threo*-3-phenylserine (L-*threo*-PS). The epimerase (E) domain, encoded in module 6, was predicted to epimerize L-*threo*-PS to D-*erythro*-PS.

The kasumigamide producer in *D*. *calyx* was identified as an ‘Entotheonella’ sp. by a single cell analysis in conjunction with PCR. This bacterial phylotype has been reported as the symbiotic producer of not only secondary metabolites in *T*. *swinhoei* [**7**], but also calyculin A in *D*. *calyx* [**13**], highlighting the fact that ‘Entotheonella’ is responsible for the production of multiple natural products in Theonellidae sponges. In addition, four kasumigamide gene clusters were detected in very different bacterial species, namely ‘Entotheonella’ sp. (a marine sponge symbiont), the free-living cyanobacterium *M*. *aruginosa* NIES-87, the human oral bacterium *D*. *acidovorans* CCUG 274B, and *Herbaspirillum* sp. CF444 from the endosphere of the tree *Populus deltoids* (**Fig 4**). Although we did not confirm the production of kasumigamide in the latter two bacterial species, the phylogenetic tree analysis of KS domains illuminate close relationships among the *kas*-related gene clusters (**S10 Fig**).

On the other hand, some peculiar features of the domain or module organization can be found in the *kas* family gene clusters. One of the remarkable points is the shift of PKS module in the *M*. *aeruginosa* NIES-87 *kas*-cluster (*makasA-D*), while the positions of the PKS module in the other *kas* genes were located on module 4, in agreement with the order of the biosynthetic reactions. To rule out the possibility that this inconsistency is due to misassembly, the contiguous domain organization between modules 5 and 6 in *makasC* was confirmed by cloning of the corresponding region. To obtain further evidence for the biosynthetic mechanism of *makasA-D*, we conducted substrate specificity assays with all five A domains, which have the substrate binding sites closely related to their counterpart domains in KasA-C with 77%~89% identity (**S2 Table**). Four of them accepted the substrates expected from the amino acid sequences of their substrate binding sites. Although the putative substrate of MakasC-A2 was L-Phe, this A domain exhibited specificity for DL-*threo*-PS. Considering the fact that D-*erythro*-PS is the C-terminal residue of kasumigamide, the biosynthetic mechanism of this step was proposed, as follows. First, L-Phe is hydroxylated by MakasD to generate L-*threo*-PS. Subsequently, MakasC-A2 loads the L-*threo*-PS onto the PCP domain. Finally, L-*threo*-PS is epimerized to D-*erythro*-PS by MakasC-E1, which is encoded between module 4 and 5 (**S13 Fig**) [**26–27**].

Other unusual features of the *kas* family genes are the absence of a thioesterase (TE) domain and the likely termination of the chain by a condensation (C) domain. Since some C domains have been reported to function as a TE domain [**28**], we expect that the C-terminal C domain encoded in *kasC*, *dakasC*, and *hkasC* can serve as a thioesterase. However, since the C-terminal C domain is missing in *makasA-D*, the release mechanism of the *makas* pathway remains unclear.

The presence of putative kasumigamide biosynthetic gene clusters among different kinds of bacteria living in various environments implies horizontal gene transfer between different bacterial species. The pair of long terminal repeats flanking the *kas* gene was annotated as putative transposases (**Table 1**, **S14 Fig**), suggesting the role of transposons in interspecies transfer of *kas* gene clusters. Sponge-associated bacteria reportedly contain high numbers of transposable insertion elements, expected to take part in the evolution of symbiont bacteria genomes [**29**]. Examples of hypothetical horizontal transmission have been suggested for gene clusters encoding synthesis of actin-binding macrolides, such as luminolide, tolytoxin, and misakinolide [**8**]. Although macrolide compounds are produced by different bacterial species, including ‘Entotheonella serta’ (which is associated with the marine sponge *T*. *swinhoei* WA), the gene clusters encoding some transposases exhibit high relationship between the corresponding PKS domains [**8**]. The *makas* gene is also flanked by two putative transposases, ORFM1 and ORFM2, which are widely conserved in several *M*. *aeruginosa* strains. Notably, *makasA-D* sequences were only found in *M*. *aeruginosa* NIES-87 strain (**S8C Fig**) among sixteen different strains whose genomes are available in National Center for Biotechnology Information (NCBI) data bank. As in the case for *M*. *aeruginosa* strains, only one of four sequenced *D*. *acidovorans* strains contains the *kas* family genes, though we could not observe a putative transposase region in the *kas*-related genes encoded in the other two kinds of bacterial species, the β-proteobacteria, *D*. *acidovorans* CCUG 274B and *Herbaspirillum* sp. CF444.

It is known that the same or similar secondary metabolites were identified from different kinds of bacteria, even across phyla. Lyngbyatoxins, which are potent skin irritants, were originally isolated from the marine cyanobacterium *Moorea producens* (formerly *Lyngbya majuscule*) [**30–31]**. On the other hand, structurally and pharmacologically related compounds, teleocidin and olivoretin, were isolated from the marine *Streptomyces* spp [**32–35**]. Saxitoxin, which is produced by marine dinoflagellates, is also made by some freshwater cyanobacteria [**36**]. The gene clusters were assembled independently in the distantly related bacteria [**37**]. Thus, the study presented here suggests that other bacteria may also have the ability to produce kasumigamide. Although the biological activity of kasumigamide had been reported to be antialgae against *Chlamydomonas neglecta* NIES-439 [**21**], further investigations are required to decipher its advantageous role in the survival competition among taxonomically distant bacterial species.

## Materials and Methods

### Specimen collection

The marine sponge *D*. *calyx* was collected by hand, at a depth of 10 m, during scuba diving at Shikine-jima Island, Tokyo, on May 18, 2011. The specimens were kept frozen at −30°C and used for the construction of the clone library and isolation of kasumigamide. The single cell isolation was performed with the specimen collected at a depth of 10 m in the ocean near Nakagi, Shizuoka, Japan in December 4, 2013. Samples were transported to the laboratory (4 h) in a cooling box and immediately processed for single cell analysis. Permits and approval for the collections at Shikine-jima and Nakagi were obtained from the local governments. Both specimens contained calyculins, calyxamides as well as kasumigamide, which was confirmed by LC-MS analysis.

### Fosmid clone library screening

The *D*. *calyx* metagenomic DNA fosmid library is composed of 250,000 colonies, each contains approximately 40 kb of insert DNA, as previously reported [**38**]. We focused on the DCKS10, which shares homology with the *cis*-AT-type-KS domains (the KS region of JamM [**39**], 59% identity), among 19 different partial sequences of KS domains obtained in our previous PCR analysis with KS degenerate primers from *D*. *calyx* metagenomic DNA [**10**]. The following primer pair was used: DCKS10F/DCKS10R (**S1 Table**) for screening positive fosmids. The library was screened by the Piel pooling strategy [**40**]. The screening yielded two positive fosmids, pDCYN1-2.

### Genome sequencing and assembly

The sequencing of pDCYN1-2 was performed on an Ion PGM sequencer (Life Technologies), with a total number of 48,198 sequence reads (~300 bp). These sequences were assembled using the *de novo* assembler MIRA (v3.4.2.0) [**41**], and 17 contigs were obtained. Further assembly to produce larger contigs was achieved with the Genious assembler (Biomatters), with the default medium sensitivity. This assembly provided a contig of 40 kb. Putative protein-coding sequences (CDSs) were determined by a combination of FramePlot [**42**] and the Glimmer 3.02 [**43**]. The domain organizations were assessed by BLASTP and PKS/NRPS analysis [**44**].

### Isolation and structure elucidation of kasumigamide

To isolate the putative *kasA-I* product from the frozen sponge, *D*. *calyx* (2.0 kg, wet weight), the methanol extract was partitioned between hexane and H_2_O. The aqueous layer was then partitioned between EtOAc and H_2_O. The aqueous layer was further partitioned between n-BuOH and H_2_O. The n-BuOH-soluble material was fractionated by gel-filtration column chromatography (Sephadex LH-20; 2.5 × 75 cm) with MeOH. The fraction was further separated by HPLC (Cosmosil MS-II C18; 10 × 250 mm, Nacalai Tesque, flow rate 4.0 mL/min; 0−100% CH_3_CN/H_2_O over 30 min; UV detection at 280 nm) to obtain pure kasumigamide (2.3 mg). The LC-MS data for monitoring were obtained from an Agilent 1100 series HPLC-micro TOF mass spectrometer (Bruker Daltonics), using electrospray ionization with a Cosmosil 5C_18_ MS-II column (2.0 × 75 mm), 5–100% MeOH/H_2_O in 0.1% AcOH over 20 min, 0.2 mL/min, positive ESI mode. To monitor an (M + H)^+^ ion peak of kasumigamide at *m/z* 787.38, the mass range between *m/z* 787.3 and 787.5 was selected for the extracted ion chromatogram.

### PCR analysis of dissected filamentous bacterial cells

Aliquots of the calcium/magnesium-free artificial seawater suspension of minced sponge tissues were spread onto Membrane slides (PEN-Membrane 2.0 μm, Leica), dehydrated by sequential incubations in 50%, 70%, and 90% aqueous ethanol for 3 min at each step, and air dried. Two or ten portions of autofluorescent cells and single filament or five filaments of filamentous bacteria (‘Entotheonella’ sp.) were directly isolated into a PCR tube by laser microdissection (Leica LMD7000). As other bacteria in **Fig 3B**, the membrane area containing other bacterial cells, except for filamentous bacterial cells, was concurrently dissected. The template DNA for the dissection PCR was adjusted according to a previously published procedure [**13**]. The PCR was performed in a volume of 10 μl, containing 1.75 mM MgCl_2_, 0.4 μM of each primers, 0.3 mM dNTPs and 0.25 U of KAPATaq Extra DNA polymerase (Nippon Genetics). The specific primers DCKS10F/DCKS10R were used for detection of the kasumigamide biosynthetic gene cluster.

### Metabolic and genomic analysis of *M*. *aeruginosa* NIES-87

*M*. *aeruginosa* NIES-87 was obtained from the National Institute for Environmental Studies collection, and cultured in MA medium [**45**]. The methanol extract (9.6 mg) of freeze-dried bacteria was analyzed on LC-MS, as described above. The *M*. *aeruginosa* NIES-87 genomic DNA was isolated according to the previously published method [**46**]. Sequencing of the genomic DNA was performed by an Ion PGM sequencer, with a total number of 414,749 sequence reads (~300 bp). These sequences were assembled *de novo* into contigs by using the Genious assembler, with the default medium sensitivity. Putative CDSs were determined by combining the prediction results from the FramePlot [**42**] and the Glimmer 3.02 [**43**] programs into one large contig (26 kb). The domain organizations were assessed by BLASTP and PKS/NRPS analysis [**44**]. In order to confirm the DNA sequence of *makasC*, the cloning of the corresponding region was performed with the primer pair, MA5F/MA6R (**S1 Table**). The PCR products amplified from the *M*. *aeruginosa* NIES-87 genomic DNA were introduced into pT7Blue T-vector (Novagen). The constructed plasmid pMAYN1 was subjected to the sequence analysis by Eurofins Genomics K. K.

### Heterologous expression of adenylation domains

To examine the substrate specificity of the A domains, we performed the heterologous expression of five A domains: MakasA-A1, MakasB-A1, MakasB-A2, MakasC-A1, and MakasC-A2. Although MakasA-A1, MakasB-A2, and MakasC-A1 were expressed as the single A domain, MakasB-A1 and MakasC-A2 were coexpressed with the corresponding C domain and PCP domain in *E*. *coli*. The DNA fragments encoding *makasA-A1*, *makasB-A1*, and *makasC-A2* were amplified by PCR and cloned into pCold II plasmid (Takara). The DNA fragments encoding *makasB-A2*, *makasC-A1* were amplified by PCR and cloned into NhisMBP-pET28b(+). In NhisMBP-pET28b(+), MBP was introduced into the N-terminal region of the pET28b(+) (Novagen) multicloning site. The *makasA-A1*, *makasB-A1*, *makasB-A2*, *makasC-A1*, and *makasC-A2* sequences were amplified with the MakasA-A1F/MakasA-A1R, MakasB-A1F/MakasB-A1R, MakasB-A2F/MakasB-A2R, MakasC-A1F/MakasC-A1R, and MakasC-A2F/MakasC-A2R primers, respectively (**S1 Table**). The *E*. *coli* BLR(DE3) cells harboring the plasmids pCold II *makasA-A1*/*makasB-A1*/*makasC-A2* and NhisMBP-pET28b(+) *makasB-A2*/*makasC-A1* were cultured at 37°C to an OD_600_ of 0.6–0.8, in LB medium, containing 100 μg/ml ampicillin or 50 μg/ml kanamycin. To induce protein expression, 0.5 mM isopropyl-1-thio-β-D-galactopyranoside was added to the cooled cultures. Expression was performed at 15°C for 16 h. All of the following procedures were performed at 4°C. For MakasA-A1, MakasB-A1, and MakasC-A2, the cells were collected by centrifugation at 10,000 *g* and resuspended in 50 mM Tris-HCl buffer (pH 8.0), containing 300 mM NaCl, 10% (v/v) glycerol and 5 mM imidazole (buffer A). The cells were disrupted by sonication, and the lysate was centrifuged at 10,000 *g* for 20 min. The supernatant was loaded onto a COSMOGEL His-Accept (Nacalai Tesque) column equilibrated with buffer A. After washing the resin with buffer A containing 10 mM imidazole, Nhis-MakasA-A1, Nhis-MakasB-A1, and Nhis-MakasC-A2 were eluted with buffer A containing 300 mM imidazole. For MakasB-A2 and MakasC-A1, the cells were collected by centrifugation at 10,000 *g* and resuspended in 50 mM Tris-HCl buffer (pH 8.0), containing 300 mM NaCl, 10% (v/v) glycerol (buffer B). The cells were disrupted by sonication, and the lysate was centrifuged at 10,000 *g* for 20 min. The supernatant was loaded onto an amylose resin (BioLabs) column equilibrated with buffer B. After washing the resin with buffer B, NhisMBP-MakasB-A2 and NhisMBP-MakasC-A1 were eluted with buffer B containing 20 mM maltose. These eluate were filtered, using an Amicon Ultra 10 K device (Merck Millipore), to remove the imidazole and to concentrate the protein.

### Functional analysis of adenylation domains

Each purified protein (1 μM) was incubated with 1 mM substrate in 50 μl of buffer, containing 32 mM hydroxylamine, 1 mM dithiothreitol, 0.4 U/ml pyrophosphatase (Sigma), 0.5 mM ATP, 10 mM MgCl_2_, and 50 mM Tris-HCl buffer (pH7.5). The reaction was incubated for 10 min at room temperature, and then quenched by adding 50 μl of the working reagent from the malachite green phosphate assay kit (Enzo). After a 10 min incubation at room temperature, the absorption at 620 nm (A_620_) was measured. The control A_620_ value was subtracted from the A_620_ value of the reaction mixture, and then the relative adenylation activity was calculated.

## Supporting Information

S1 Fig ESI-TOFMS of kasumigamide. (TIFF)

S2 Fig ^1^H NMR spectrum of kasumigamide in DMSO-*d*_6_ + TFA. (TIFF)

S3 Fig ^1^H-^1^H-COSY spectrum of kasumigamide in DMSO-*d*_6_ + TFA. (TIFF)

S4 Fig ^13^C NMR spectrum of kasumigamide in DMSO-*d*_6_ + TFA. (TIFF)

S5 Fig HMQC spectrum of kasumigamide in DMSO-*d*_6_ + TFA. (TIFF)

S6 Fig HMBC spectrum of kasumigamide in DMSO-*d*_6_ + TFA. (TIFF)

S7 Fig LC-MS data for the *M*. *aeruginosa* NIES-87 extraction and kasumigamide. Extracted ion chromatogram (EIC) of *m/z* 787.3–787.5, derived from **(a)** *M*. *aeruginosa* NIES-87 extract and **(c)** kasumigamide isolated from *D*. *calyx*. **(b)** and **(d)** are scan mass spectra of the peaks labeled in **(a)** and **(c)**, respectively.
(TIFF)

S8 Fig ORFs encoded in the putative biosynthetic gene cluster of kasumigamide. Each gene cluster is derived from **(a)** *D*. *acidovorans* CCUG 274B **(b)** *Herbaspirillum* sp. CF444 **(c)** *M*. *aeruginosa* NIES-87. The ORFs related to PKS-NRPS are highlighted in red. The ORFs and regions, widely conserved in *M*. *aeruginosa* strains, are highlighted in green allow. Red arrow indicates the specific regions, detected only in the *M*. *aeruginosa* NIES-87 genomic DNA.
(TIFF)

S9 Fig SDS-PAGE of expressed A domains. 10% SDS-PAGE of **(a)** MakasA-A1 (61.8 kDa), **(b)** MakasB-CA1P (118.6 kDa), **(c)** MakasB-A2 (101.2 kDa), **(d)** MakasC-A1 (109.8 kDa), and **(e)** MakasC-CA2P (119.1 kDa), purified by Ni-NTA affinity chromatography.
(TIFF)

S10 Fig Phylogenetic tree analysis of KS domains. KS domains derived from *kas* family genes are highlighted in red. BarE: AAN32979, CurA: AAT70096, CurF: AAT70101, CurG: AAT70102, CurH: AAT70103, CurI: AAT70104, CurJ: AAT70105, CurK: AAT70106, CurL: AAT70107, CurM: AAT70108, EpoA: ABB92690, EpoC: ABB92692, EpoD: ABB92693, EpoE: ABB92694, EpoF: ABB92695, JamE: AAS98777, JamJ: AAS98781, JamK: AAS98782, JamL: AAS98783, JamM: AAS98784, JamP: AAS98787, MmxB: ABA29782, MmxC: ABA29781, MscA: AHB82051, MscB: AHB82052, MscC: AHB82053, MscD: AHB82054, MscG: AHB82057, MscI: AHB82059, MxaB: AAK57186, MxaC: AAK57187, MxaD: AAK57188, MxaE: AAK57189, MxaF: AAK57190, NpnA: AEU11005, NpnB: AEU11006, StiA: CAD19085, StiB: CAD19086, StiC: CAD19087, StiD: CAD19088, StiE: CAD19089, StiF: CAD19090, StiG: CAD19091, StiH: CAD19092, StiJ: CAD19093, TlmVIII: ABL74938, TtcA: AGC65513, ZmaA: ACM79805, ZmaK: AAR87760.
(TIFF)

S11 Fig Phylogenetic tree analysis of 16S rRNA. The bacteria, possessing *kas* family genes, are highlighted in red.
(TIFF)

S12 Fig Comparison of the amino acid sequences of CmlA and KasD homologs. # Residues involved in metal ion binding.
(TIFF)

S13 Fig A hypothetical scheme for constructing D-*erythro*-PS. (TIFF)

S14 Fig Long terminal repeats flanking the *kas* gene. Underlined DNA sequence region shows ORF1 or ORF2 encoding putative transposase.
(TIFF)

S1 Table PCR primers used in this study. (TIFF)

S2 Table Predicted A domain substrates. The putative substrates of A domains were predicted by the NRPS codes except for the first A domain (KasA-A1 and its homologs). The substrates of MakasA-C are indicated based on the *in vitro* assay.
(TIFF)

S3 Table NMR spectroscopic data for Kasumigamide in DMSO-*d*_6_ + TFA. (TIFF)

S4 Table Comparison of NMR spectroscopic data with literature values. Each NMR data for kasumigamide derived from the original literature (X) in DMSO-*d*_6_ and our data (Y) in DMSO-*d*_6_ + TFA.
(TIFF)

S5 Table Putative ORFs of kasumigamide NRPS-PKS gene cluster derive from *M*. *aeruginosa* NIES-87. (TIFF)

S6 Table Putative ORFs of kasumigamide NRPS-PKS gene cluster derive from *D*. *acidovorans* CCUG 274B. (TIFF)

S7 Table Putative ORFs of kasumigamide NRPS-PKS gene cluster derive from *Herbaspirillum* sp. CF444.
(TIFF)

## References

[1] Donia MS, Cimermancic P, Schulze CJ, Wieland Brown LC, Martin J, Mitreva M, et al A systematic analysis of biosynthetic gene clusters in the human microbiome reveals a common family of antibiotics. Cell. 2014; 158: 1402–1414. 10.1016/j.cell.2014.08.032 25215495 PMC4164201

[2] Fischbach MA, Walsh CT. Assembly-line enzymology for polyketide and nonribosomal peptide antibiotics: logic, machinery, and mechanisms. Chem Rev. 2006; 106: 3468–3496. 10.1021/cr0503097 16895337

[3] Kampa A, Gagunashvili AN, Gulder TA, Morinaka BI, Daolio C, Godejohann M, et al Metagenomic natural product discovery in lichen provides evidence for a family of biosynthetic pathways in diverse symbioses. Proc Natl Acad Sci U S A. 2013; 110: 3129–3137. 10.1073/pnas.1305867110 23898213 PMC3746887

[4] Hentschel U, Piel J, Degnan SM, Taylor MW. Genomic insights into the marine sponge microbiome. Nat Rev Microbiol. 2012; 10: 641–654. 10.1038/nrmicro2839 22842661

[5] Piel J, Hui D, Wen G, Butzke D, Platzer M, Fusetani N, et al Antitumor polyketide biosynthesis by an uncultivated bacterial symbiont of the marine sponge *Theonella swinhoei*. Proc Natl Acad Sci U S A. 2004; 101: 16222–16227. 10.1073/pnas.0405976101 15520376 PMC528957

[6] Freeman MF, Gurgui C, Helf MJ, Morinaka BI, Uria AR, Oldham NJ, et al Metagenome mining reveals polytheonamides as posttranslationally modified ribosomal peptides. Science. 2012; 338: 387–390. 10.1126/science.1226121 22983711

[7] Wilson MC, Mori T, Rückert C, Uria AR, Helf MJ, Takada K, et al An environmental bacterial taxon with a large and distinct metabolic repertoire. Nature. 2014; 506: 58–62. 10.1038/nature12959 24476823

[8] Ueoka R, Uria AR, Reiter S, Mori T, Karbaum P, Peters EE, et al Metabolic and evolutionary origin of actin-binding polyketides from diverse organisms. Nat Chem Biol. 2015; 11; 705–712. 10.1038/nchembio.1870 26236936 PMC7116039

[9] Döderlein L. Studien an japanischen Lithistiden. Zeitschr. f. wiss. Zool. 1883; 40: 62–104.

[10] Kato Y, Fusetani N, Matsunaga S, Hashimoto K, Fujita S, Furuya T. Bioactive marine metabolites. Part 16. Calyculin A. A novel antitumor metabolite from the marine sponge *Discodermia calyx*. J Am Chem Soc. 1986; 108: 2780–2781. 10.1021/ja00270a061

[11] Egami Y, Wakimoto T, Abe I. Phosphocalyculin C as a pyrophosphate protoxin of calyculin C in the marine sponge *Discodermia calyx*. Bioorg Med Chem Lett. 2014; 24: 5150–5153. 10.1016/j.bmcl.2014.10.002 25442302

[12] Kimura M, Wakimoto T, Egami Y, Tan KC, Ise Y, Abe I. Calyxamides A and B, cytotoxic cyclic peptides from the marine sponge *Discodermia calyx*. J Nat Prod. 2012; 75: 290–294. 10.1021/np2009187 22276742

[13] Wakimoto T, Egami Y, Nakashima Y, Wakimoto Y, Mori T, Awakawa T, et al Calyculin biogenesis from a pyrophosphate protoxin produced by a sponge symbiont. Nat Chem Biol. 2014; 10: 648–655. 10.1038/nchembio.1573 24974231

[14] Piel J, Hui D, Fusetani N, Matsunaga S. Targeting modular polyketide synthases with iteratively acting acyltransferases from metagenomes of uncultured bacterial consortia. Environ Microbiol. 2004; 8: 921–927. 10.1111/j.1462-2920.2004.00531.x 15305917

[15] Beyer S, Kunze B, Silakowski B, Muller R. Metabolic diversity in myxobacteria: identification of the myxalamid and the stigmatellin biosynthetic gene cluster of *Stigmatella aurantiaca* Sg a15 and a combined polyketide-(poly)peptide gene cluster from the epothilone producing strain *Sorangium cellulosum* So ce90. Biochim Biophys Acta. 1999; 1445: 185–195. 10.1016/s0167-4781(99)00041-x 10320771

[16] Moffitt MC, Neilan BA. Evolutionary affiliations within the superfamily of ketosynthases reflect complex pathway associations. J Mol Evol. 2003; 56: 446–457. 10.1007/s00239-002-2415-0 12664164

[17] Schirmer A, Gadkari R, Reeves CD, Ibrahim F, DeLong EF, Hutchinson CR. Metagenomic analysis reveals diverse polyketide synthase gene clusters in microorganisms associated with the marine sponge *Discodermia dissoluta*. Appl Environ Microbiol. 2005; 71: 4840–4849. 10.1128/AEM.71.8.4840-4849.2005 16085882 PMC1183291

[18] Ginolhac A, Jarrin C, Gillet B, Robe P, Pujic P, Tuphile K, et al Phylogenetic analysis of polyketide synthase I domains from soil metagenomic libraries allows selection of promising clones. Appl Environ Microbiol. 2004; 70: 5522–5527. 10.1128/AEM.70.9.5522-5527.2004 15345440 PMC520897

[19] Stachelhaus T, Mootz HD, Marahiel MA. The specificity-conferring code of adenylation domains in nonribosomal peptide synthetases. Chem Biol. 1999; 6: 493–505. 10.1016/S1074-5521(99)80082-9 10421756

[20] Ishida K, Welker M, Christiansen G, Cadel-Six S, Bouchier C, Dittmann E, et al Plasticity and evolution of aeruginosin biosynthesis in cyanobacteria. Appl Environ Microbiol. 2009; 75: 2017–2026. 10.1128/AEM.02258-08 19201978 PMC2663223

[21] Ishida K, Murakami M. Kasumigamide, an antialgal peptide from the cyanobacterium *Microcystis aeruginosa*. J Org Chem. 2000; 65: 5898–5900. 10.1021/jo991918f 10987919

[22] McQuade TJ, Shallop AD, Sheoran A, Delproposto JE, Tsodikov OV, Garneau-Tsodikova S. A nonradioactive high-throughput assay for screening and characterization of adenylation domains for nonribosomal peptide combinatorial biosynthesis. Anal Biochem. 2009; 386: 244–250. 10.1016/j.ab.2008.12.014 19135023

[23] Kadi N, Challis GL. Chapter 17. Siderophore biosynthesis a substrate specificity assay for nonribosomal peptide synthetase-independent siderophore synthetases involving trapping of acyl-adenylate intermediates with hydroxylamine. Methods Enzymol. 2009; 458: 431–457. 10.1016/S0076-6879(09)04817-4 19374993

[24] Brown SD, Utturkar SM, Klingeman DM, Johnson CM, Martin SL, Land ML, et al Twenty-one genome sequences from Pseudomonas species and 19 genome sequences from diverse bacteria isolated from the rhizosphere and endosphere of *Populus deltoides*. J Bacteriol. 2012; 194: 5991–5993. 10.1128/JB.01243-12 23045501 PMC3486089

[25] Makris TM, Knoot CJ, Wilmot CM, Lipscomb JD. Structure of a dinuclear iron cluster-containing β-hydroxylase active in antibiotic biosynthesis. Biochemistry. 2013; 52: 6662–6671. 10.1021/bi400845b 23980641 PMC3826434

[26] Ross AC, Xu Y, Lu L, Kersten RD, Shao Z, Al-Suwailem AM, et al Biosynthetic multitasking facilitates thalassospiramide structural diversity in marine bacteria. J Am Chem Soc. 2015; 135: 1155–1162. 10.1021/ja3119674 PMC3563429 23270364

[27] Kevany BM, Rasko DA, Thomas MG. Characterization of the complete zwittermicin A biosynthetic gene cluster from *Bacillus cereus*. Appl Environ Microbiol. 2009; 75: 1144–1155. 10.1128/AEM.02518-08 19098220 PMC2643575

[28] Du L, Lou L. PKS and NRPS release mechanisms. Nat Prod Rep. 2010; 27: 255–278. 10.1039/b912037h 20111804

[29] Thomas T, Rusch D, DeMaere MZ, Yung PY, Lewis M, Halpern A, et al Functional genomic signatures of sponge bacteria reveal unique and shared features of symbiosis. ISME J. 2010; 4: 1557–1567. 10.1038/ismej.2010.74 20520651

[30] Cardellina JH II, Marner FJ, Moore RE. Seaweed dermatitis: structure of lyngbyatoxin A. Science. 1979; 2014: 193–195. 10.1126/science.107586 107586

[31] Aimi N, Odaka H, Sakai S, Fujiki H, Suganuma M, Moore RE, et al Lyngbyatoxins B and C, two new irritants from *Lyngbya majuscule*. J Nat Prod. 1990; 53: 1593–1596. 10.1021/np50072a035 2128518

[32] Fujiki H, Mori M, Nakayasu M, Terada M, Sugimura T, Moore RE. Indole alkaloids: dihydroteleocidin B, teleocidin, and lyngbyatoxin A as members of a new class of tumor promoters. Proc Natl Acad Sci U S A. 1981; 78: 3872–3876. 10.1073/pnas.78.6.3872 6791164 PMC319675

[33] Hitotsuyanagi Y, Fujiki H, Suganuma M, Aimi N, Sakai S, Endo Y, et al Isolation and structure elucidation of teleocidin B-1, B-2, B-3, and B-4. Chem Pharm Bull (Tokyo). 1984; 32: 4233–4236. 10.1248/cpb.32.4233 6442218

[34] Hitotsuyanagi Y, Yamaguchi K, Ogata K, Aimi N, Sakai S, Koyama Y, et al Elucidation of the structures of olivoretin B and C. Chem Pharm Bull (Tokyo). 1984; 32: 3774–3778. 10.1248/cpb.32.3774 6525665

[35] Ninomiya M, Fujiki H, Paik NS, Hakii H, Suganuma M, Hitotsuyanagi Y, et al Des-O-methylolivoretin C is a new member of the teleocidin class of tumor promoters. Jpn J Cancer Res. 1986; 77: 222–225. 3084411

[36] Wiese M, D-Agostino PM, Mihali TK, Moffitt MC, Neilan BA. Neurotoxic alkaloids: saxitoxin and its analogs. Mar Drugs. 2010; 8: 2185–2211. 10.3390/md8072185 20714432 PMC2920551

[37] Hackett JD, Wisecaver JH, Brosnahan ML, Kulis DM, Anderson DM, Bhattacharya D, et al Evolution of saxitoxin synthesis in cyanobacteria and dinoflagellates. Mol Biol Evol. 2013; 30: 70–78. 10.1093/molbev/mss142 22628533 PMC3525144

[38] He R, Wakimoto T, Takeshige Y, Egami Y, Kenmoku H, Ito T, et al Porphyrins a metagenomic library of the marine sponge *Discodermia calyx*. Mol Biosyst. 2012; 8: 2334–2338. 10.1039/c2mb25169h 22735778

[39] Edwards DJ, Marquez BL, Nogle LM, McPhail K, Goeger DE, Roberts MA, et al Structure and biosynthesis of the jamaicamides, new mixed polyketide-peptide neurotoxins from the marine cyanobacterium *Lyngbya majuscula*. Chem Biol. 2004; 11: 817–833. 10.1016/j.chembiol.2004.03.030 15217615

[40] Hrvatin S, Piel J. Rapid isolation of rare clones from highly complex DNA libraries by PCR analysis of liquid gel pools. J Microbiol Methods. 2007; 68: 434–436. 10.1016/j.mimet.2006.09.009 17055603

[41] Chevreux B, Wetter T, Suhai S. Genome sequence assembly using trace signals and additional sequence information. In: Computer Science and Biology: Proceedings of the German Conference on Bioinformatics. Braunschweig (Germany): GBF Braunschweig Department of Bioinformatics. 1999; 45–56.

[42] Ishikawa J, Hotta K. FramePlot: a new implementation of the Frame analysis for predicting protein-coding regions in bacterial DNA with a high G+C content. FEMS Microbiol Lett. 1999; 174: 251–253. 10.1111/j.1574-6968.1999.tb13576.x 10339816

[43] Delcher AL, Harmon D, Kasif S, White O, Salzberg SL. Improved microbial gene identification with GLIMMER. Nucleic Acids Res. 1999; 27: 4636–4641. 10.1093/nar/27.23.4636 10556321 PMC148753

[44] Bachmann BO, Ravel J. Methods for in silico prediction of microbial polyketide and nonribosomal peptide biosynthetic pathways from DNA sequence data. Methods Enzymol. 2009; 458: 181–217. 10.1016/S0076-6879(09)04808-3 19374984

[45] Ichimura T. In: Nishizawa K, Chihara M (Eds.). 2. Isolation and culture methods of algae. 2.5.B. Freshwater algae [2. Sôrui no bunri to baiyôhô. 2.5.B. Tansui sôrui]. In: Methods in Phycological Studies [Sôrui Kenkyûhô]. Kyoritu Syuppan. 1979; 294–305, (in Japanese).

[46] Morin N, Vallaeys T, Hendrickx L, Natalie L, Wilmotte A. An efficient DNA isolation protocol for filamentous cyanobacteria of the genus *Arthrospira*. J Microbiol. 2010; Methods 80: 148–154. 10.1016/j.mimet.2009.11.012 20004220

