# Supplementary figures and images for: Metagenomic Analysis of the Sponge Discodermia Reveals the Production of the Cyanobacterial Natural Product Kasumigamide by ‘Entotheonella’
Source: PLoS One. 2016 Oct 12;11(10):e0164468. doi: 10.1371/journal.pone.0164468 (PMC5061366; doi:10.1371/journal.pone.0164468)

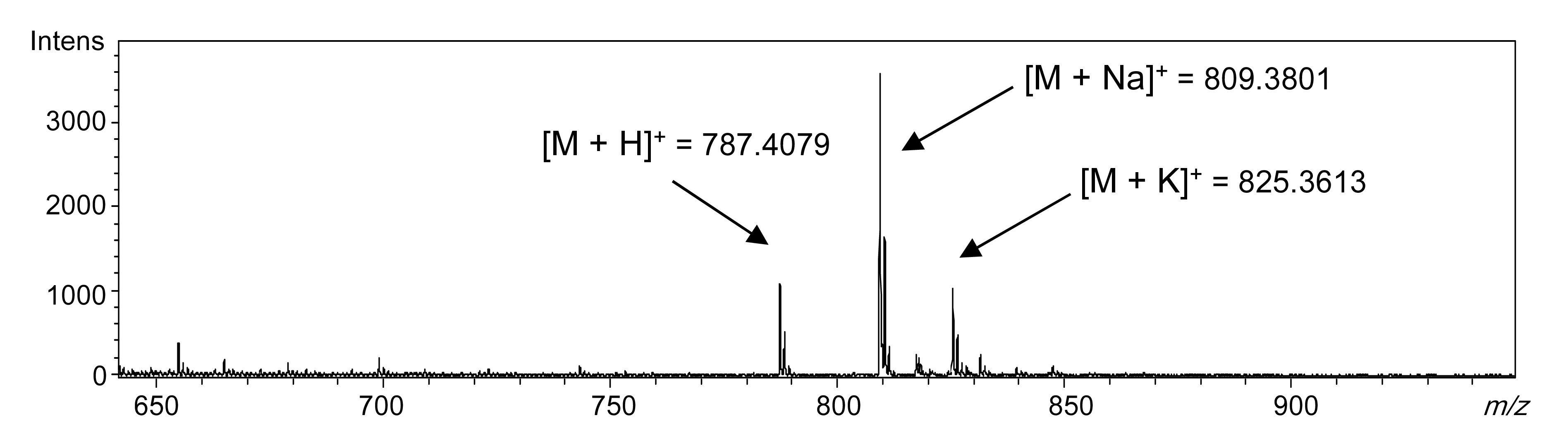

Supplement: S1 Fig — (TIFF) [file pone.0164468.s001.tiff]

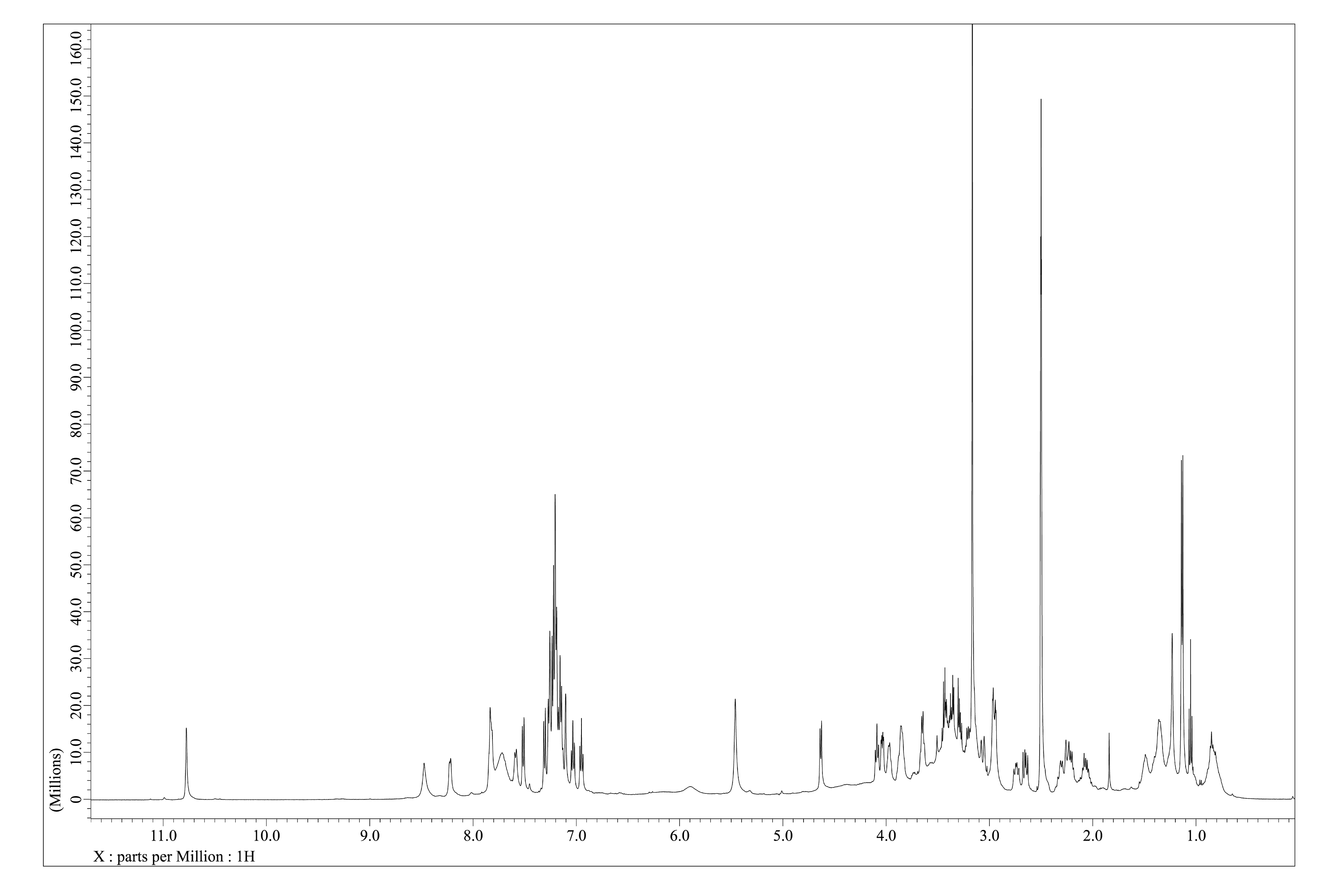

Supplement: S2 Fig — (TIFF) [file pone.0164468.s002.tiff]

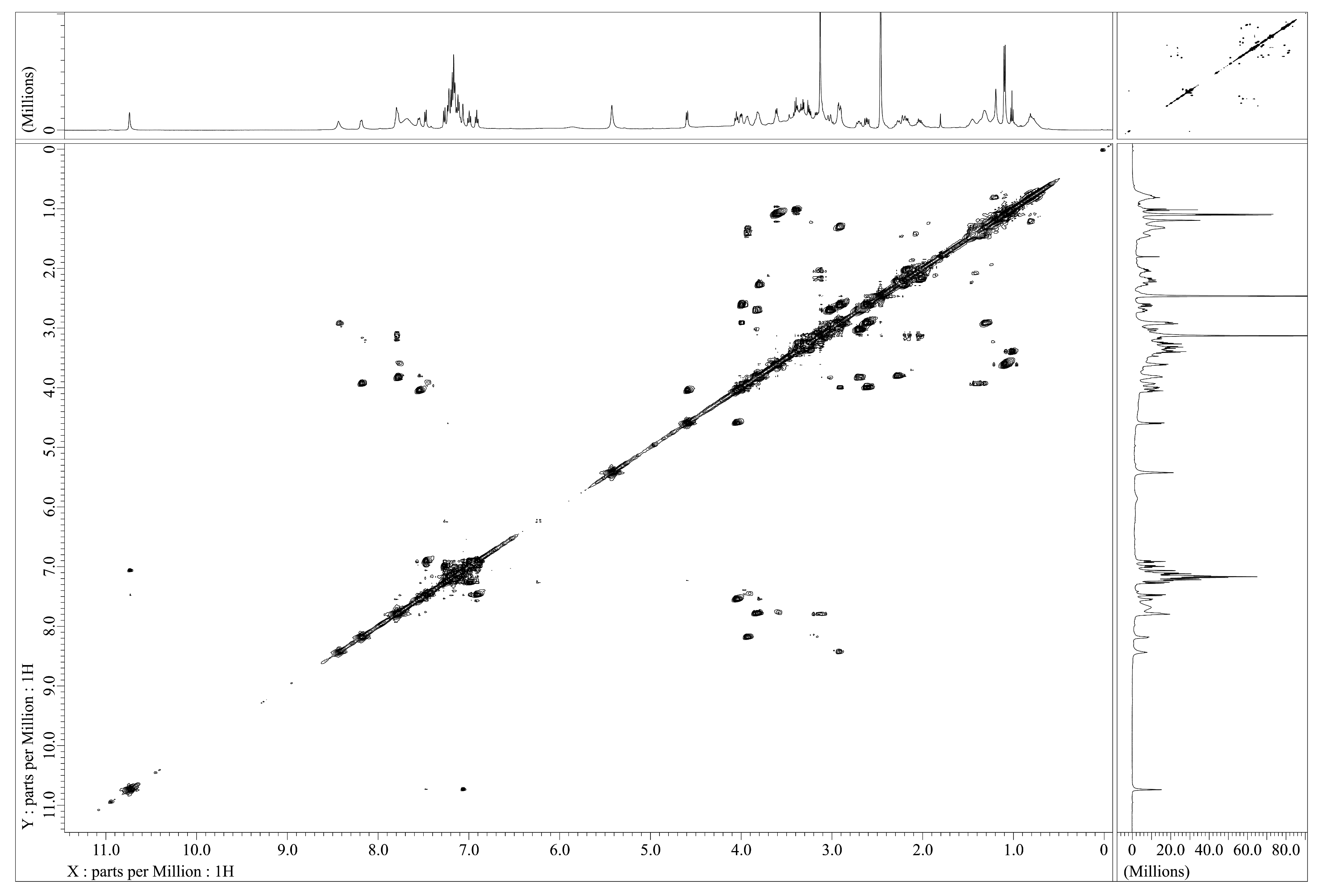

Supplement: S3 Fig — (TIFF) [file pone.0164468.s003.tiff]

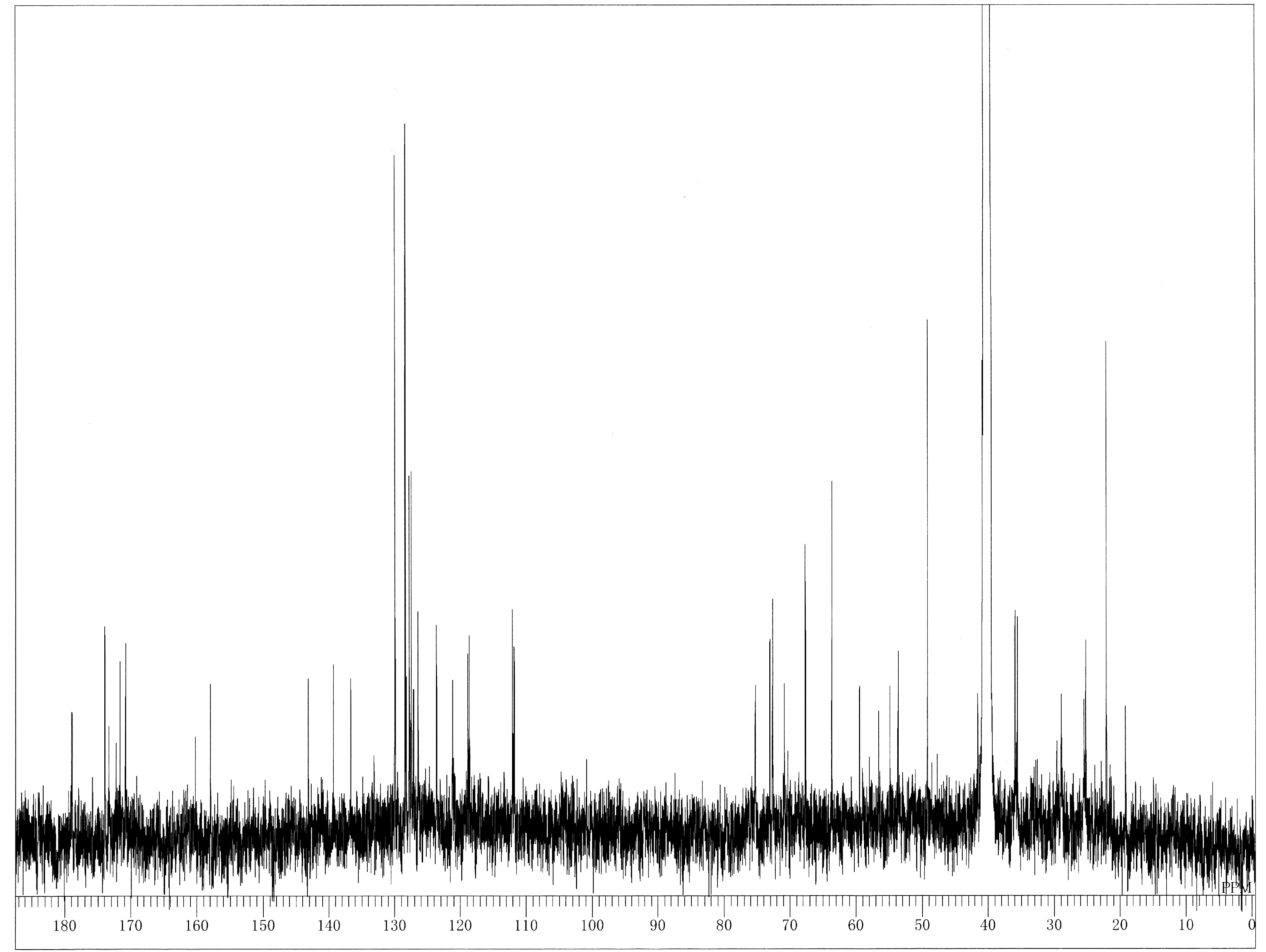

Supplement: S4 Fig — (TIFF) [file pone.0164468.s004.tiff]

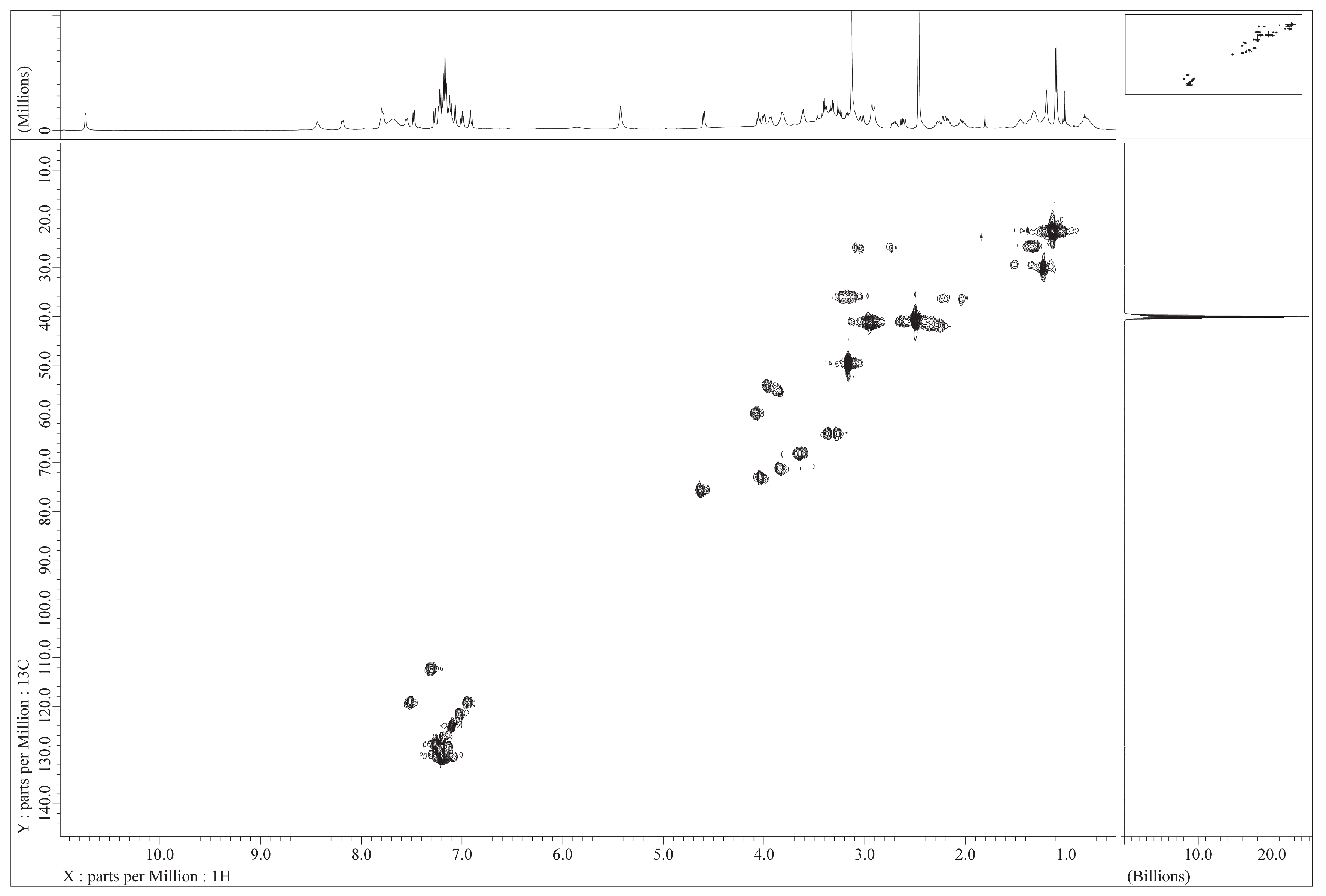

Supplement: S5 Fig — (TIFF) [file pone.0164468.s005.tiff]

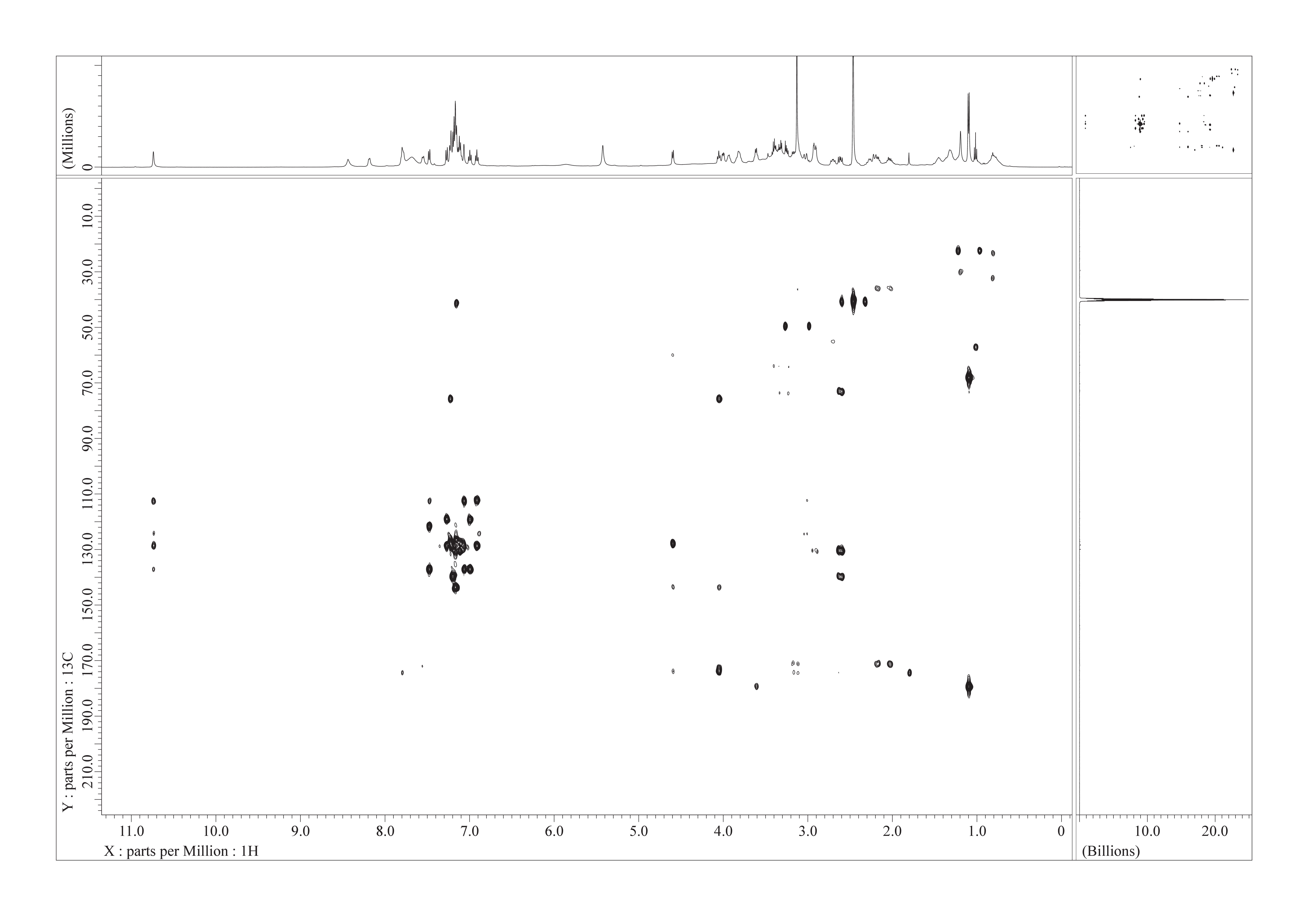

Supplement: S6 Fig — (TIFF) [file pone.0164468.s006.tiff]

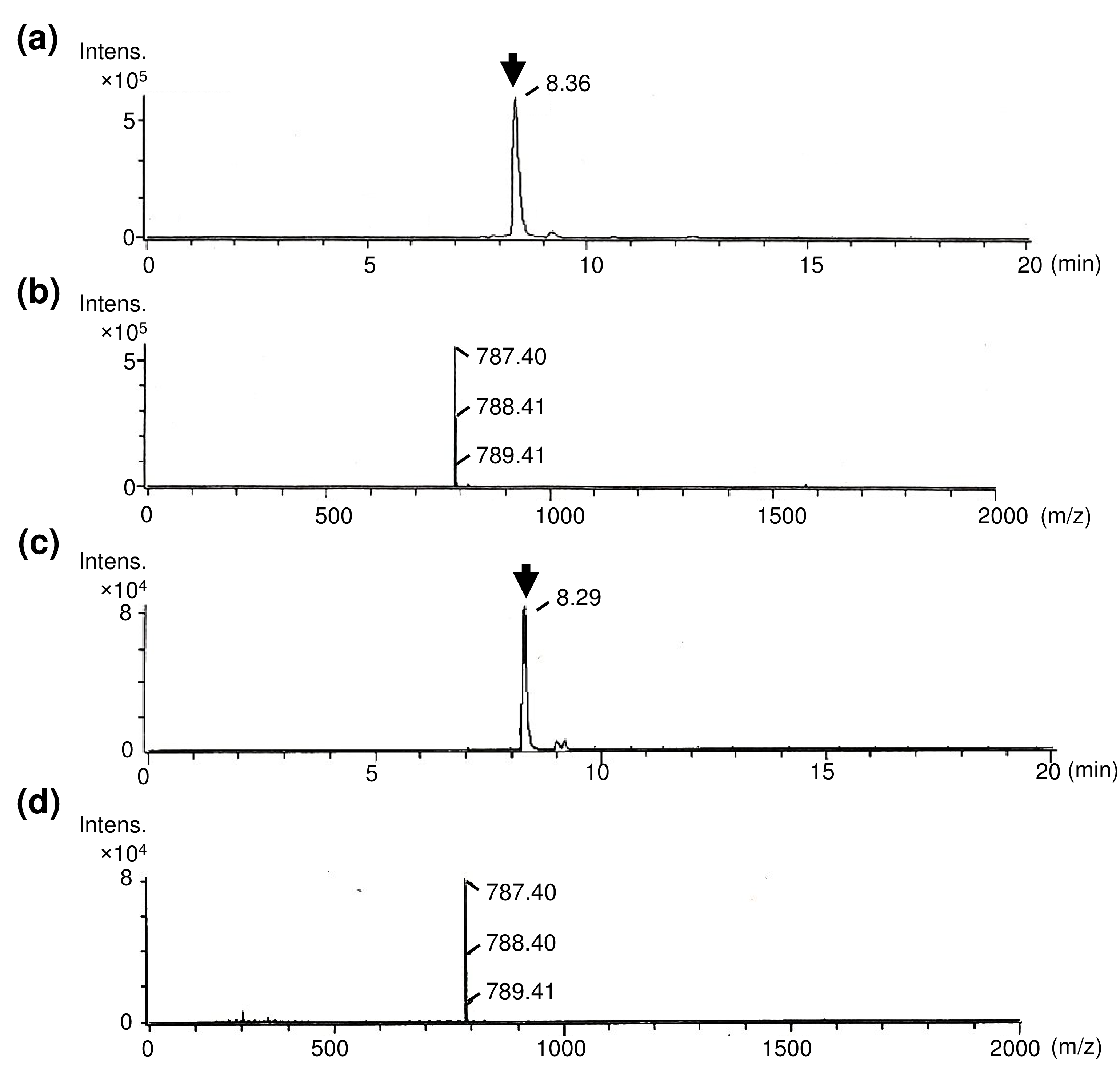

Supplement: S7 Fig — Extracted ion chromatogram (EIC) of m/z 787.3–787.5, derived from (a) M. aeruginosa NIES-87 extract and (c) kasumigamide isolated from D. calyx. (b) and (d) are scan mass spectra of the peaks labeled in (a) and (c), respectively. (TIFF) [file pone.0164468.s007.tiff]

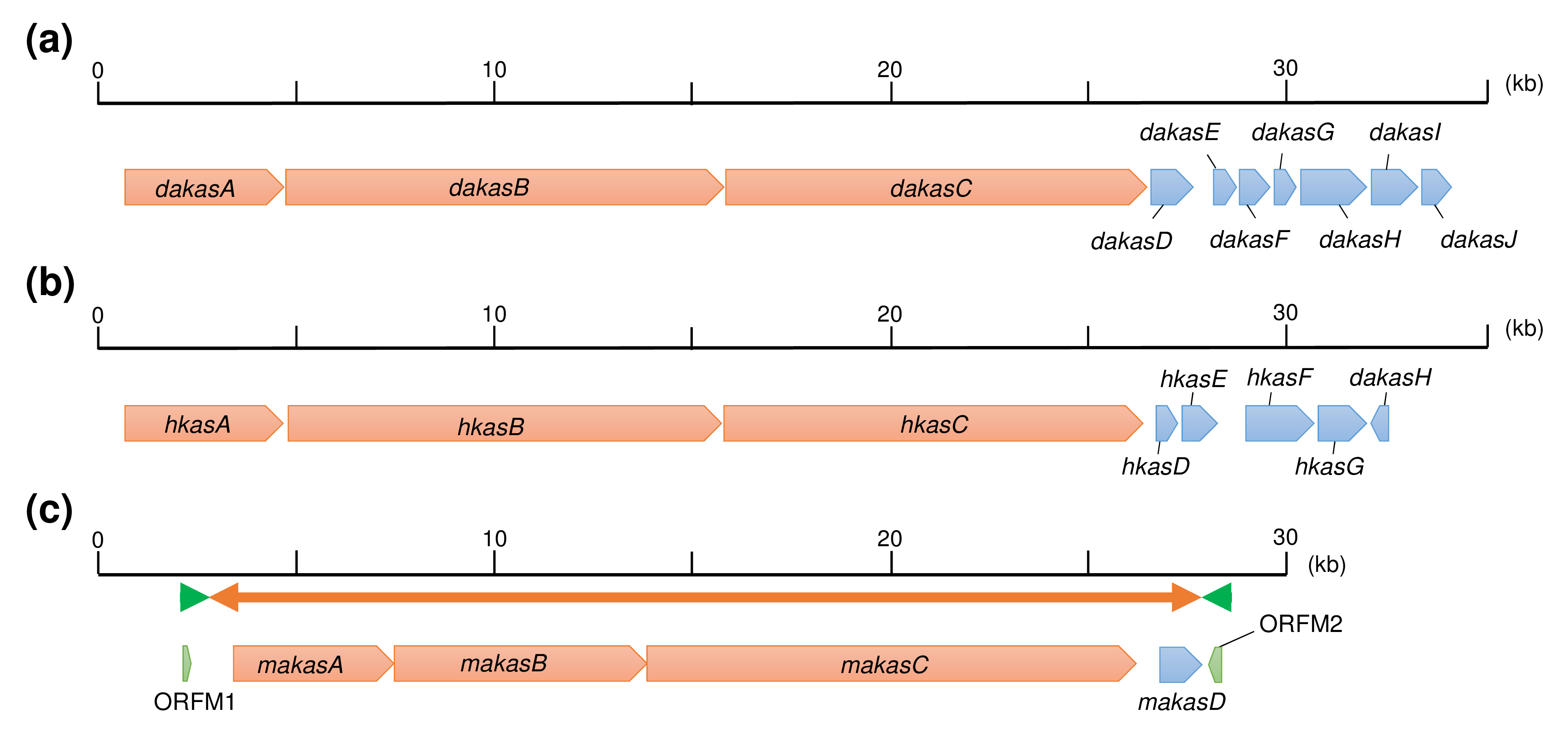

Supplement: S8 Fig — Each gene cluster is derived from (a) D. acidovorans CCUG 274B (b) Herbaspirillum sp. CF444 (c) M. aeruginosa NIES-87. The ORFs related to PKS-NRPS are highlighted in red. The ORFs and regions, widely conserved in M. aeruginosa strains, are highlighted in green allow. Red arrow indicates the specific regions, detected only in the M. aeruginosa NIES-87 genomic DNA. (TIFF) [file pone.0164468.s008.tiff]

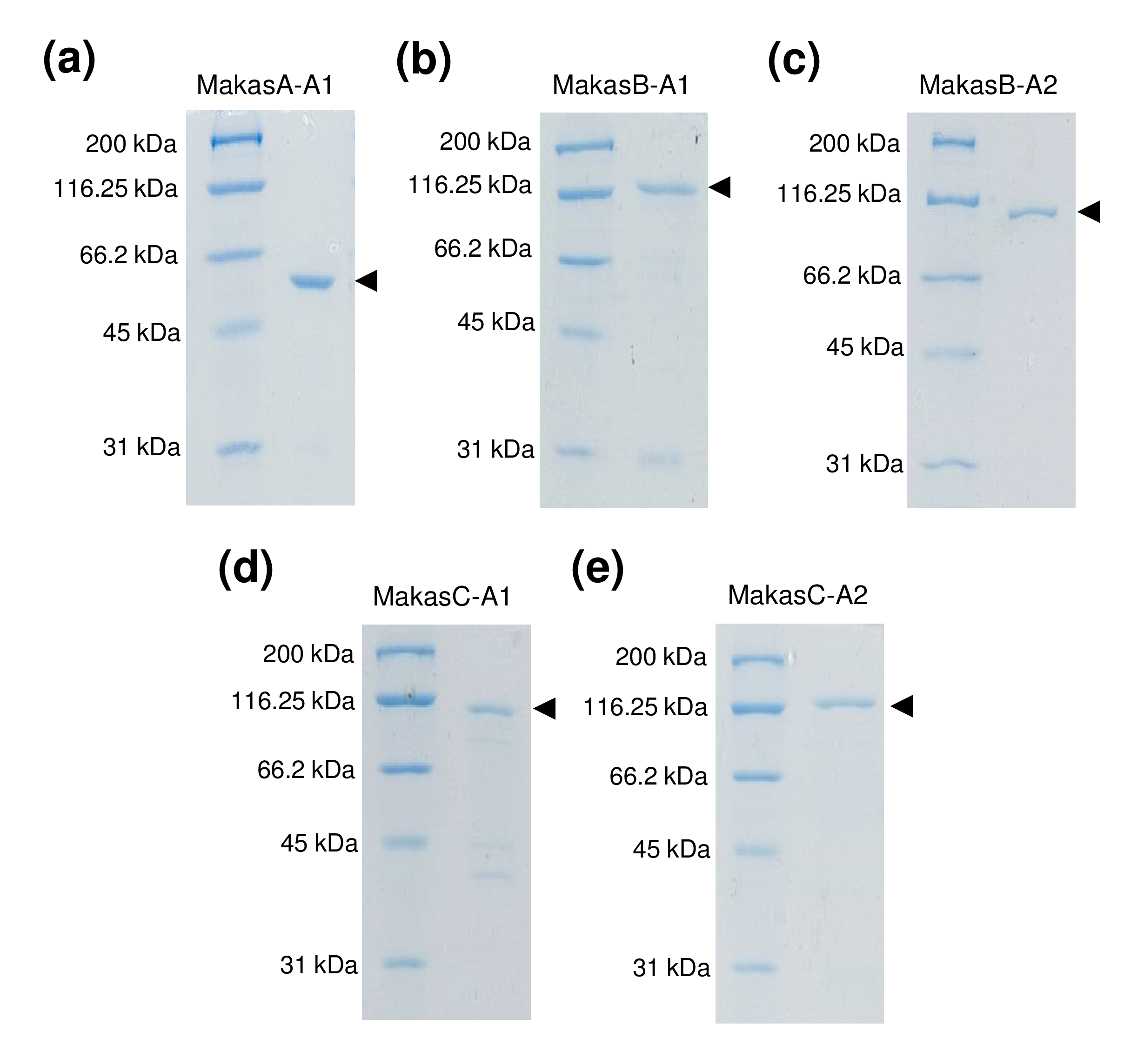

Supplement: S9 Fig — 10% SDS-PAGE of (a) MakasA-A1 (61.8 kDa), (b) MakasB-CA1P (118.6 kDa), (c) MakasB-A2 (101.2 kDa), (d) MakasC-A1 (109.8 kDa), and (e) MakasC-CA2P (119.1 kDa), purified by Ni-NTA affinity chromatography. (TIFF) [file pone.0164468.s009.tiff]

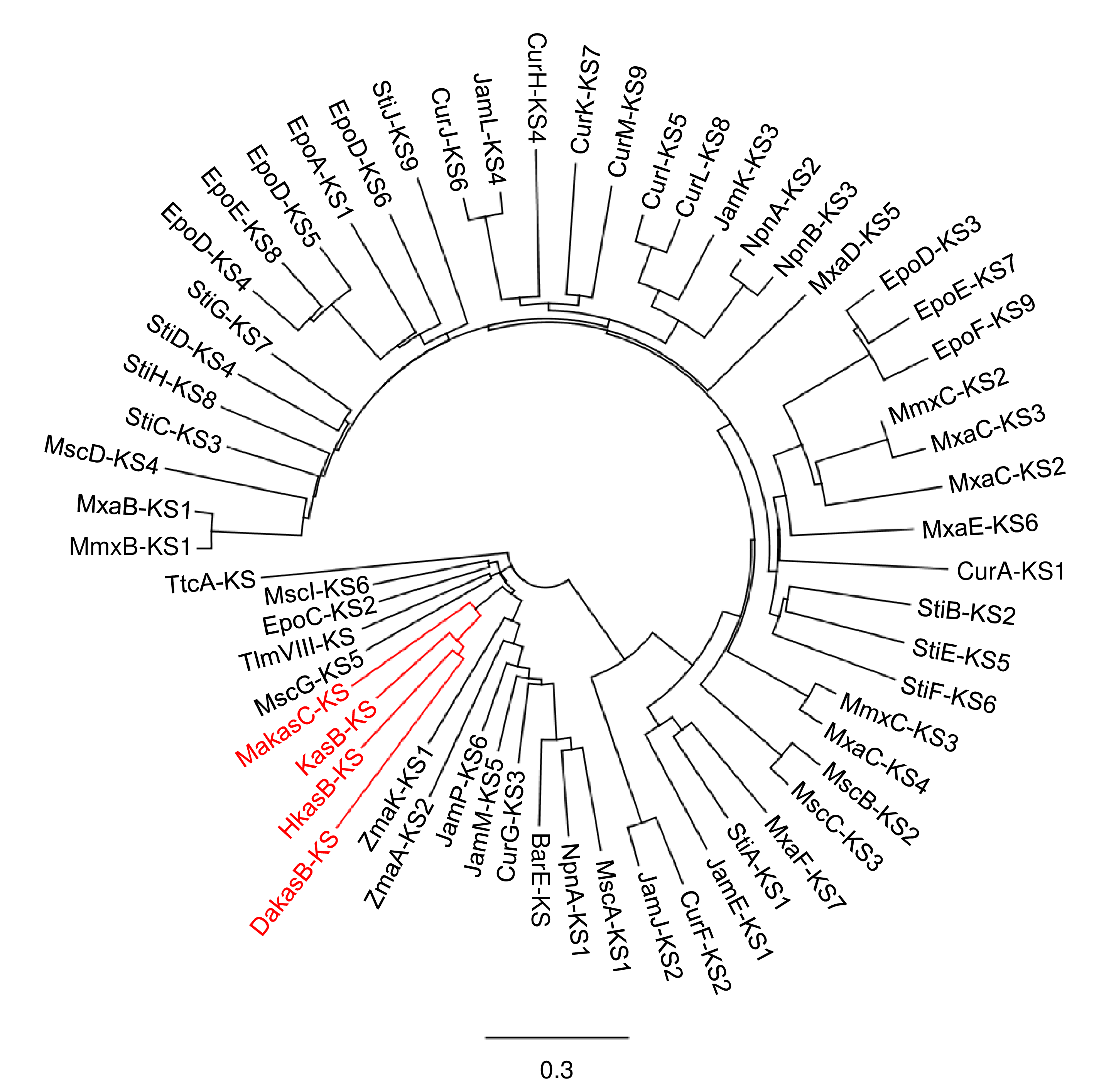

Supplement: S10 Fig — KS domains derived from kas family genes are highlighted in red. BarE: AAN32979, CurA: AAT70096, CurF: AAT70101, CurG: AAT70102, CurH: AAT70103, CurI: AAT70104, CurJ: AAT70105, CurK: AAT70106, CurL: AAT70107, CurM: AAT70108, EpoA: ABB92690, EpoC: ABB92692, EpoD: ABB92693, EpoE: ABB92694, EpoF: ABB92695, JamE: AAS98777, JamJ: AAS98781, JamK: AAS98782, JamL: AAS98783, JamM: AAS98784, JamP: AAS98787, MmxB: ABA29782, MmxC: ABA29781, MscA: AHB82051, MscB: AHB82052, MscC: AHB82053, MscD: AHB82054, MscG: AHB82057, MscI: AHB82059, MxaB: AAK57186, MxaC: AAK57187, MxaD: AAK57188, MxaE: AAK57189, MxaF: AAK57190, NpnA: AEU11005, NpnB: AEU11006, StiA: CAD19085, StiB: CAD19086, StiC: CAD19087, StiD: CAD19088, StiE: CAD19089, StiF: CAD19090, StiG: CAD19091, StiH: CAD19092, StiJ: CAD19093, TlmVIII: ABL74938, TtcA: AGC65513, ZmaA: ACM79805, ZmaK: AAR87760. (TIFF) [file pone.0164468.s010.tiff]

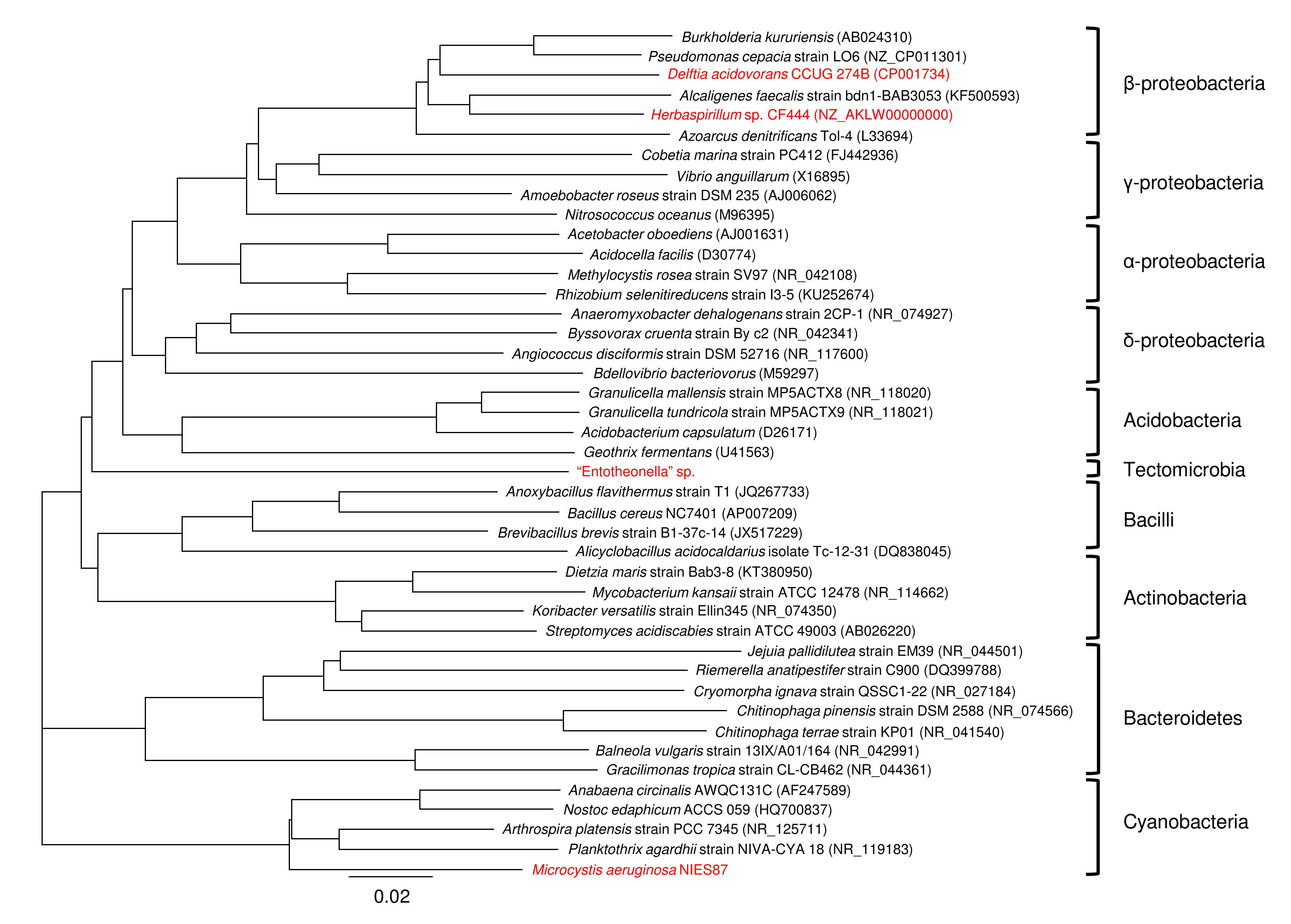

Supplement: S11 Fig — The bacteria, possessing kas family genes, are highlighted in red. (TIFF) [file pone.0164468.s011.tiff]

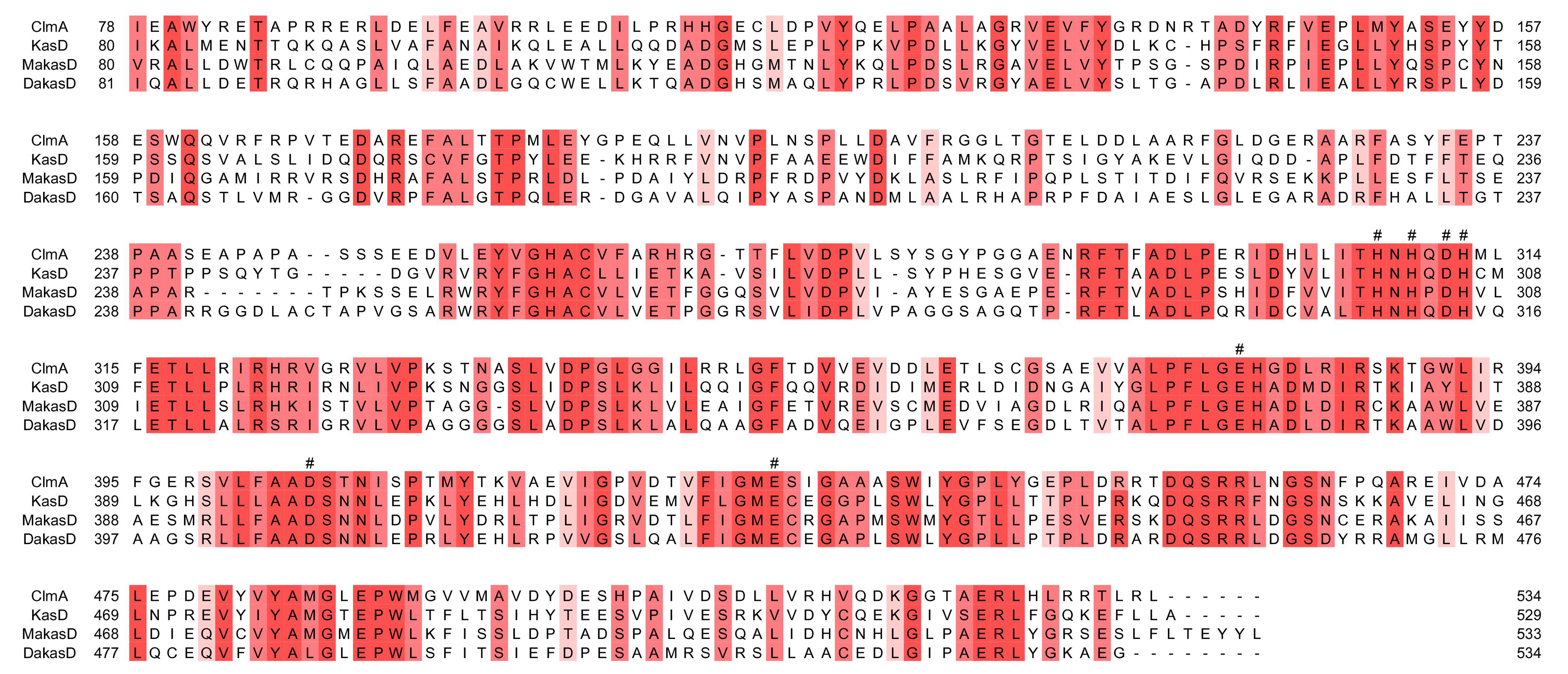

Supplement: S12 Fig — # Residues involved in metal ion binding. (TIFF) [file pone.0164468.s012.tiff]

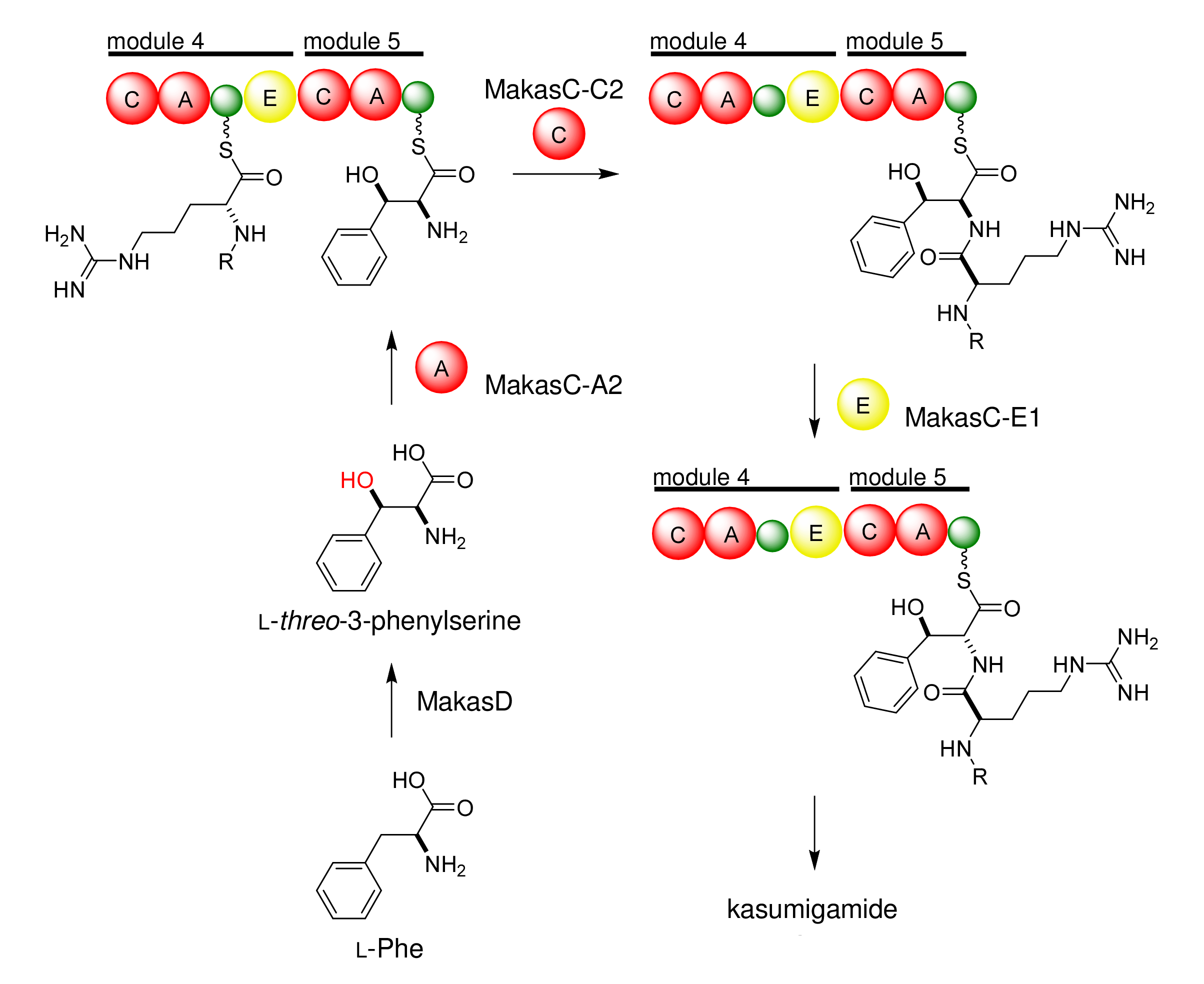

Supplement: S13 Fig — (TIFF) [file pone.0164468.s013.tiff]

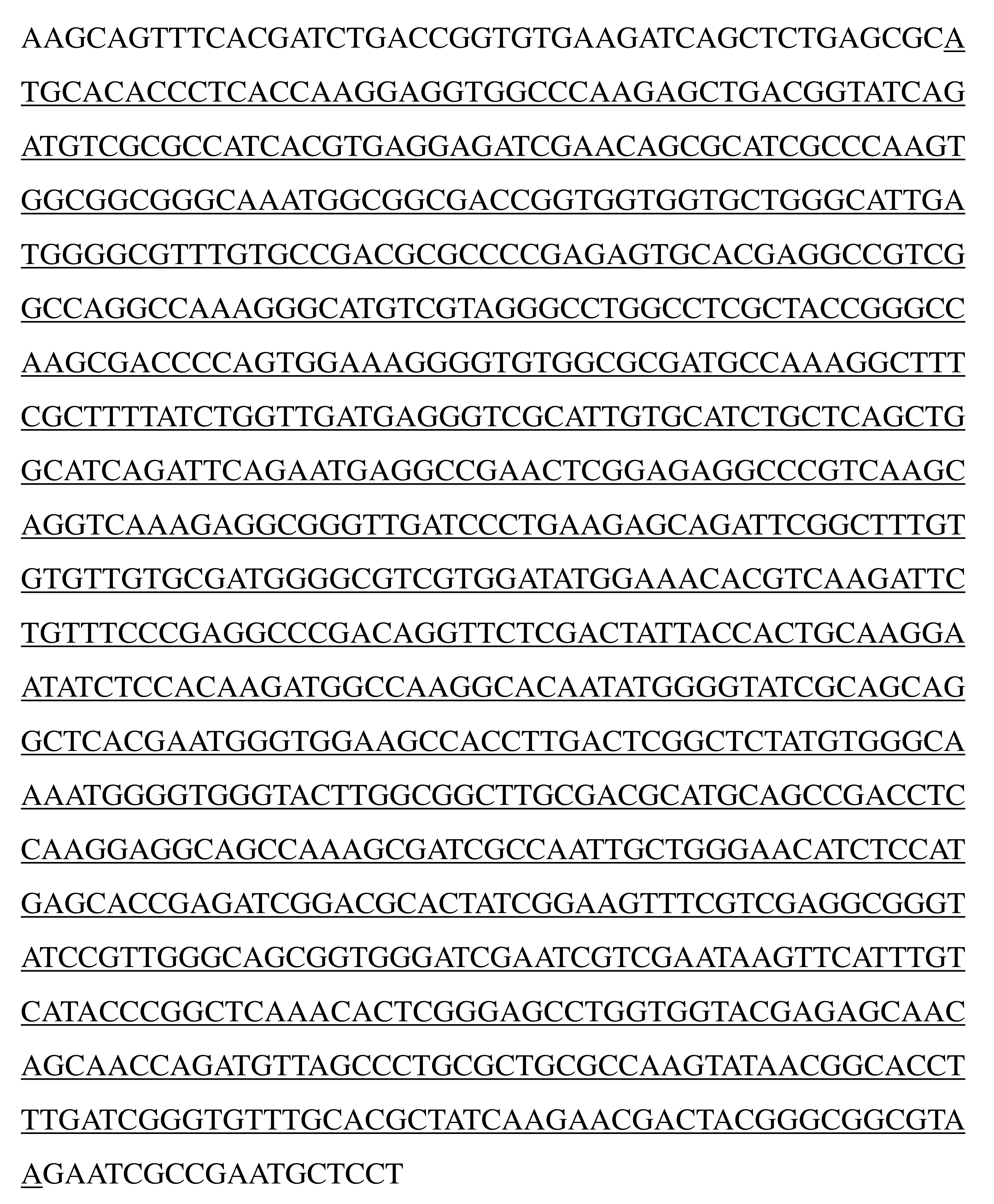

Supplement: S14 Fig — Underlined DNA sequence region shows ORF1 or ORF2 encoding putative transposase. (TIFF) [file pone.0164468.s014.tiff]

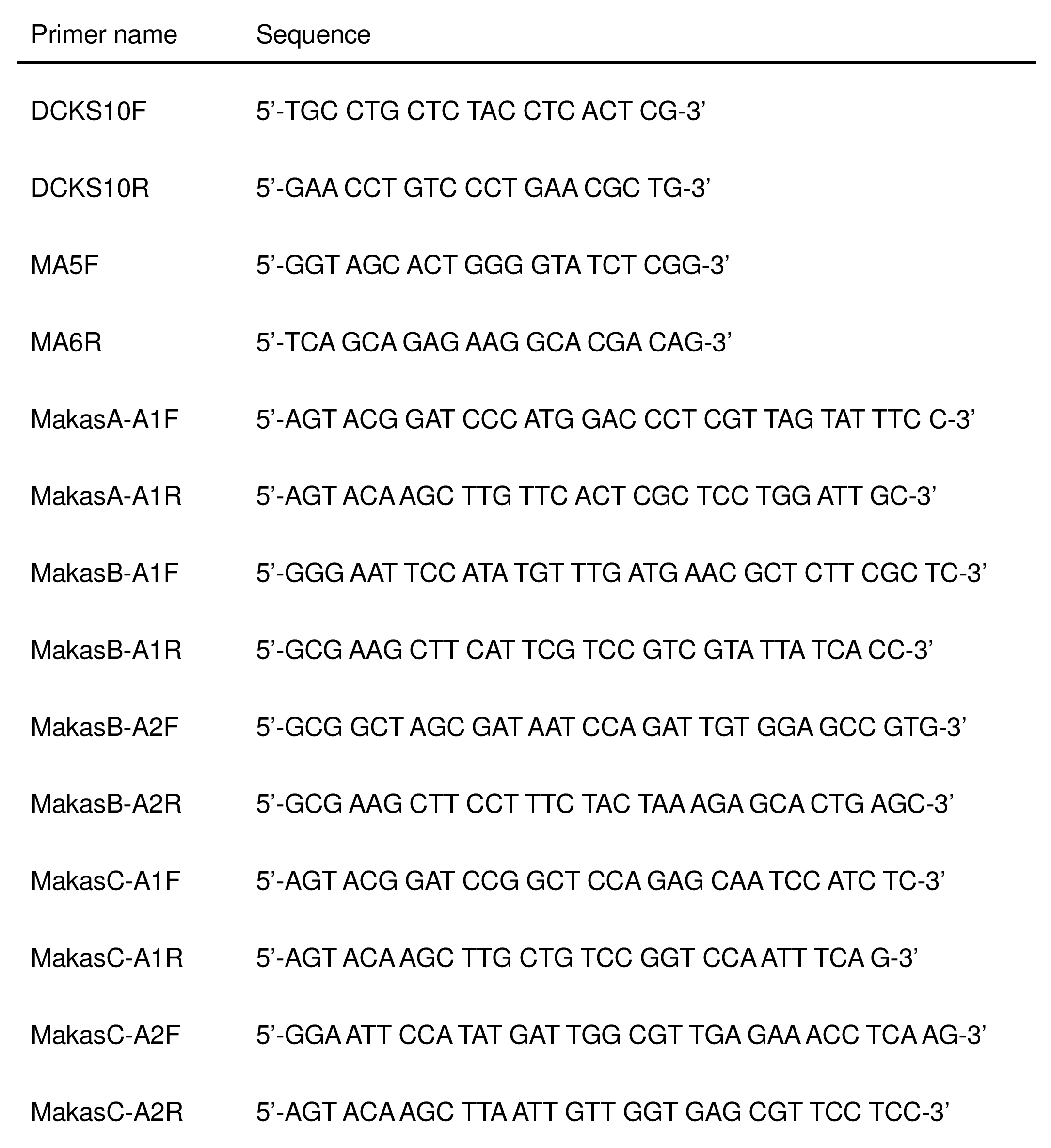

Supplement: S1 Table — (TIFF) [file pone.0164468.s015.tiff]

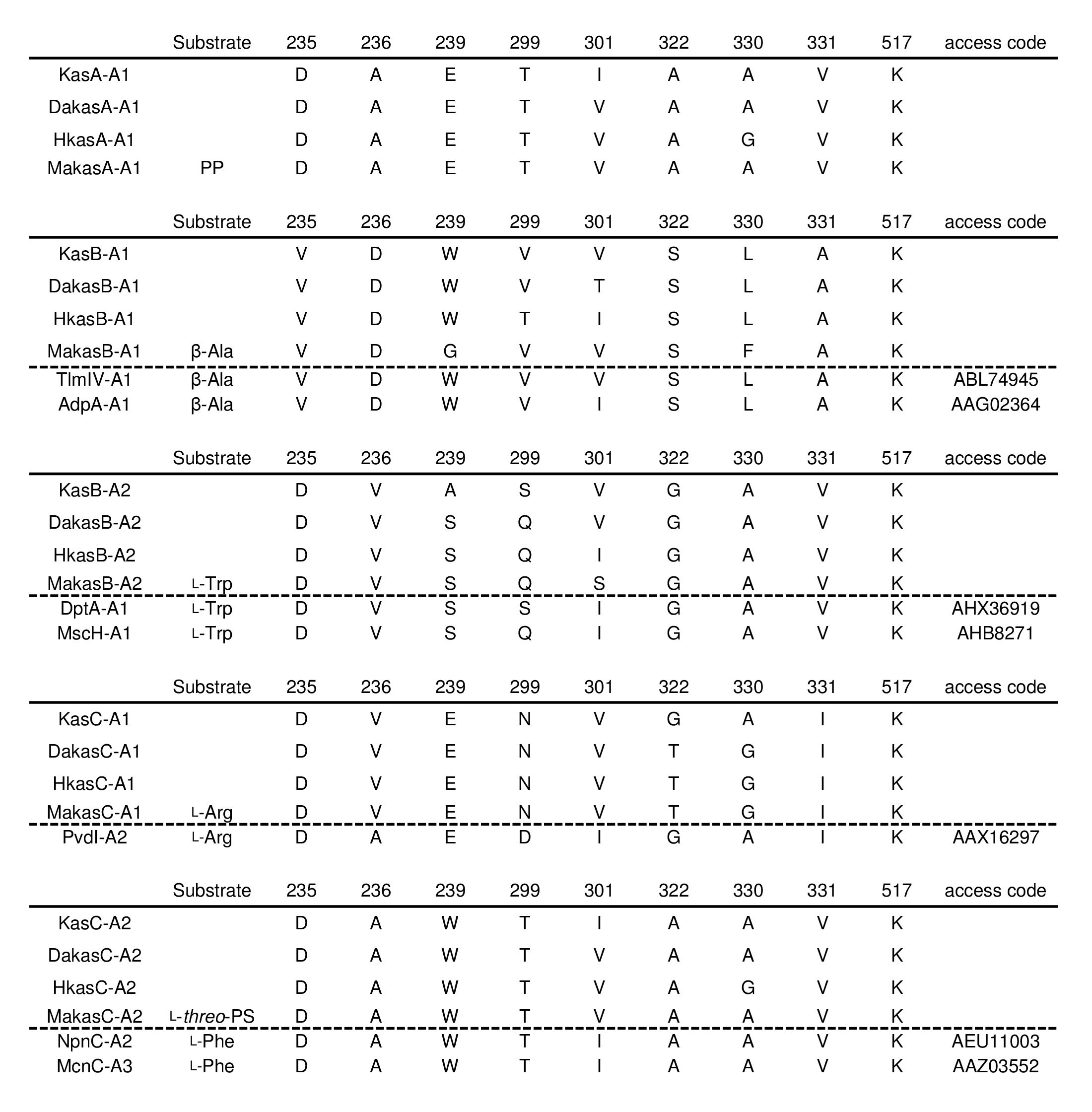

Supplement: S2 Table — The putative substrates of A domains were predicted by the NRPS codes except for the first A domain (KasA-A1 and its homologs). The substrates of MakasA-C are indicated based on the in vitro assay. (TIFF) [file pone.0164468.s016.tiff]

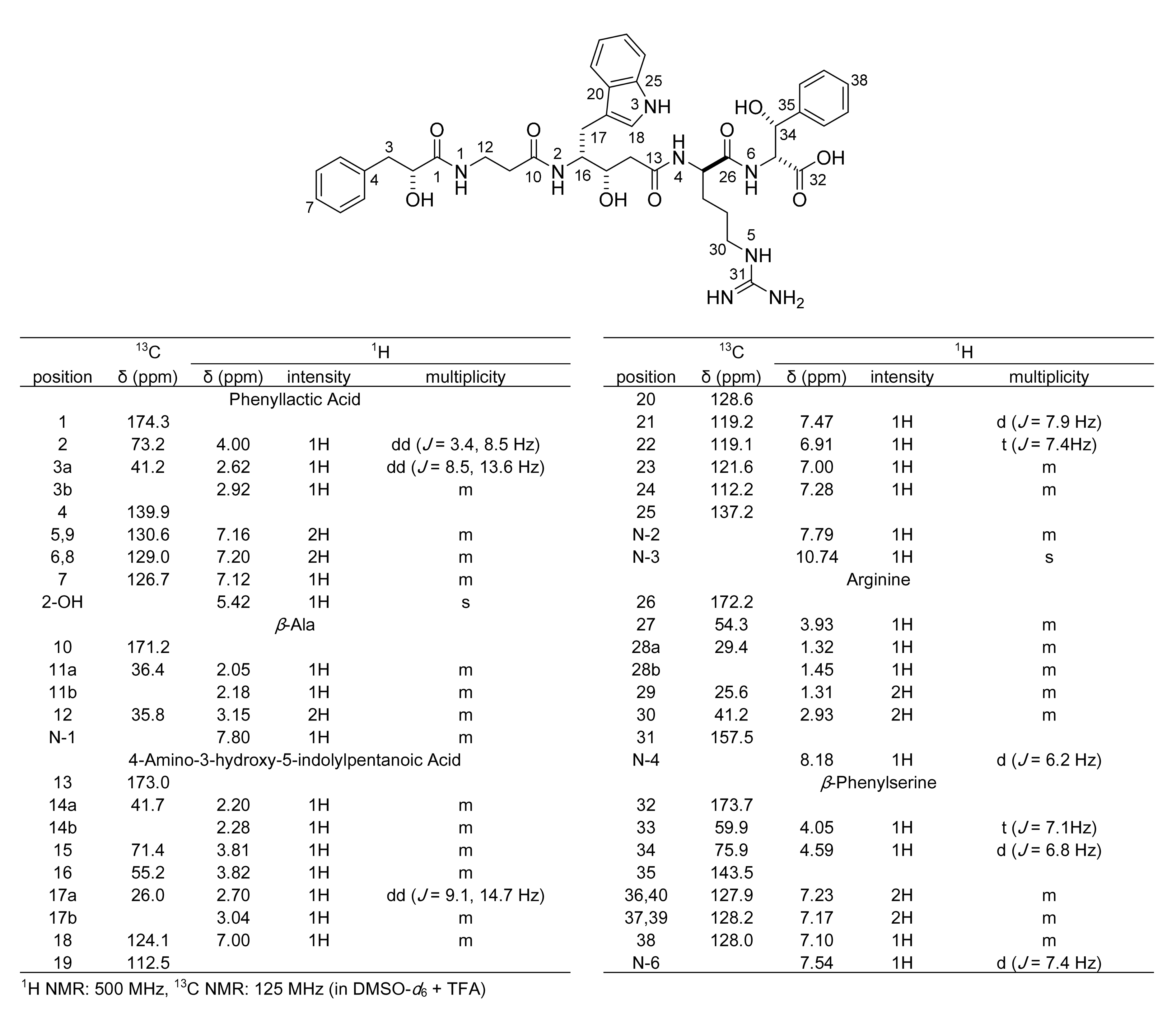

Supplement: S3 Table — (TIFF) [file pone.0164468.s017.tiff]

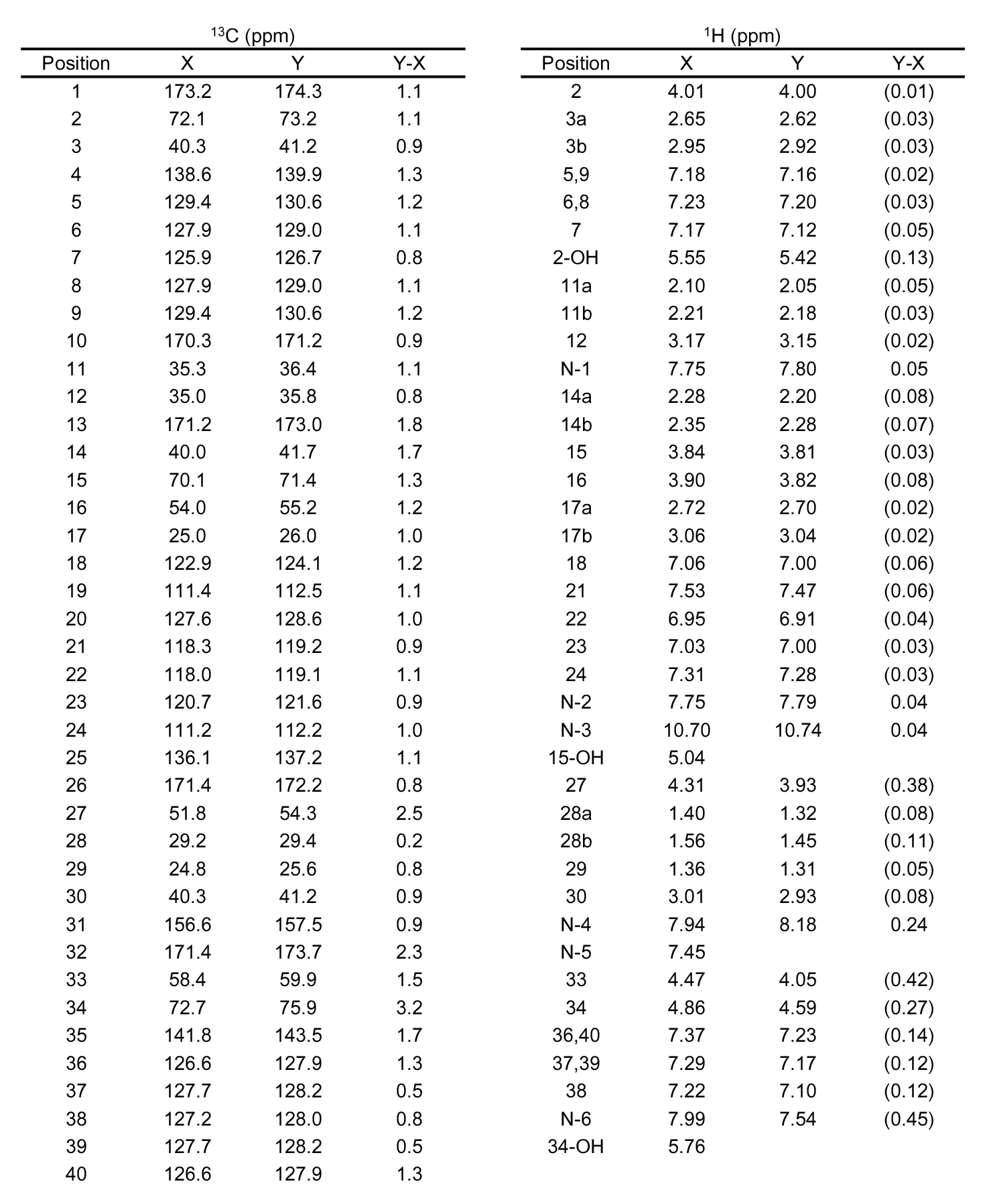

Supplement: S4 Table — Each NMR data for kasumigamide derived from the original literature (X) in DMSO-d6 and our data (Y) in DMSO-d6 + TFA. (TIFF) [file pone.0164468.s018.tiff]

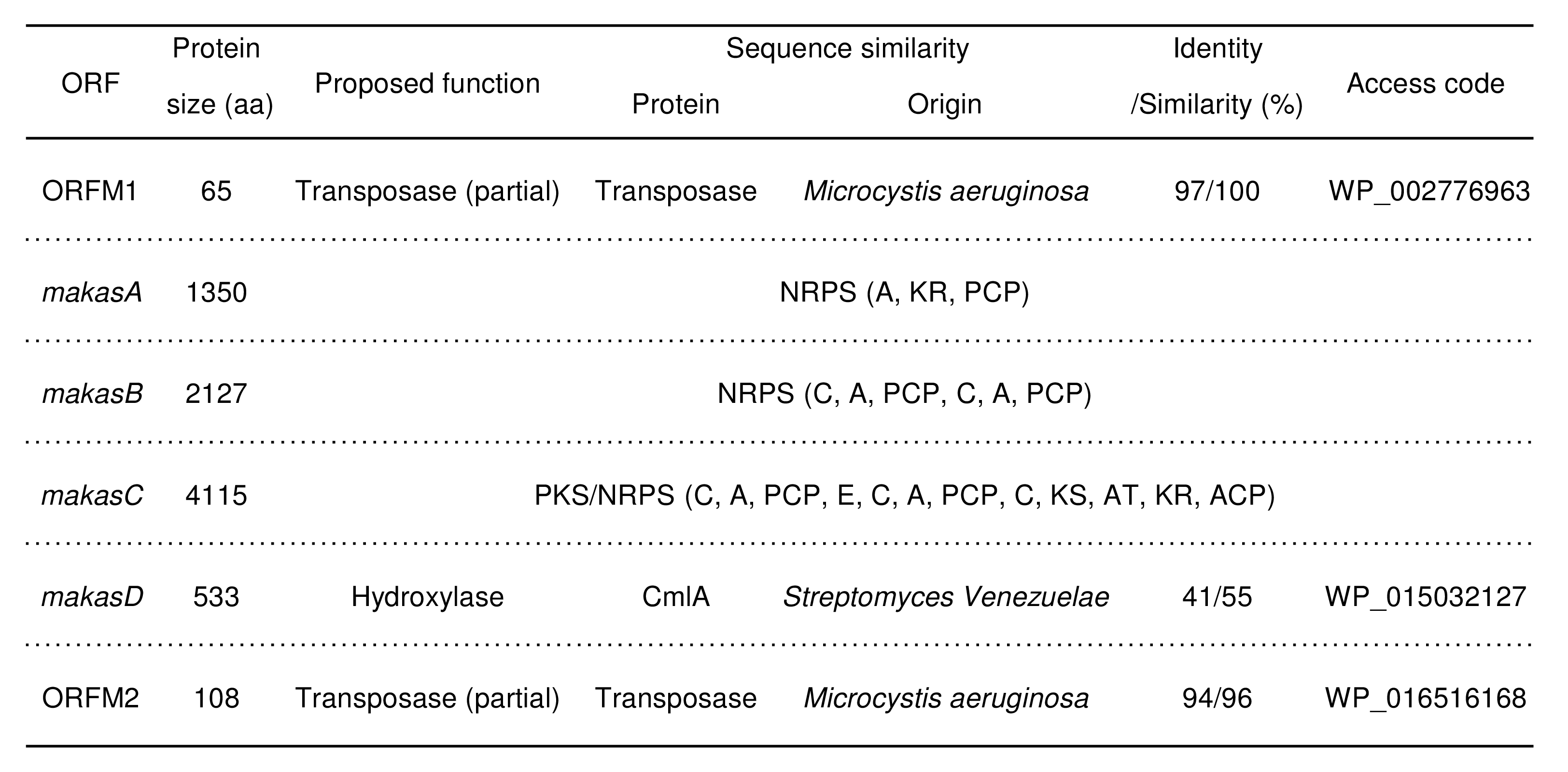

Supplement: S5 Table — (TIFF) [file pone.0164468.s019.tiff]

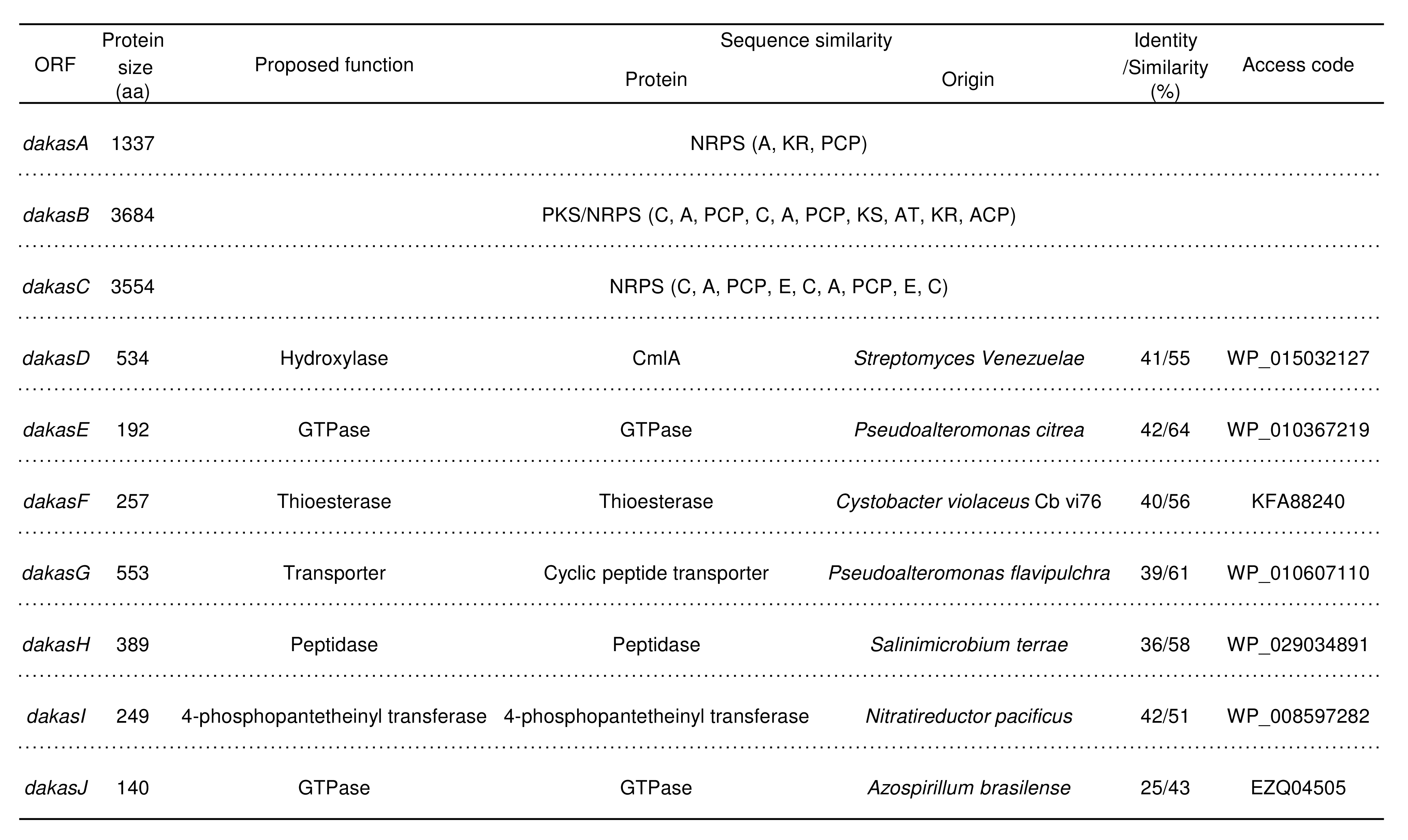

Supplement: S6 Table — (TIFF) [file pone.0164468.s020.tiff]

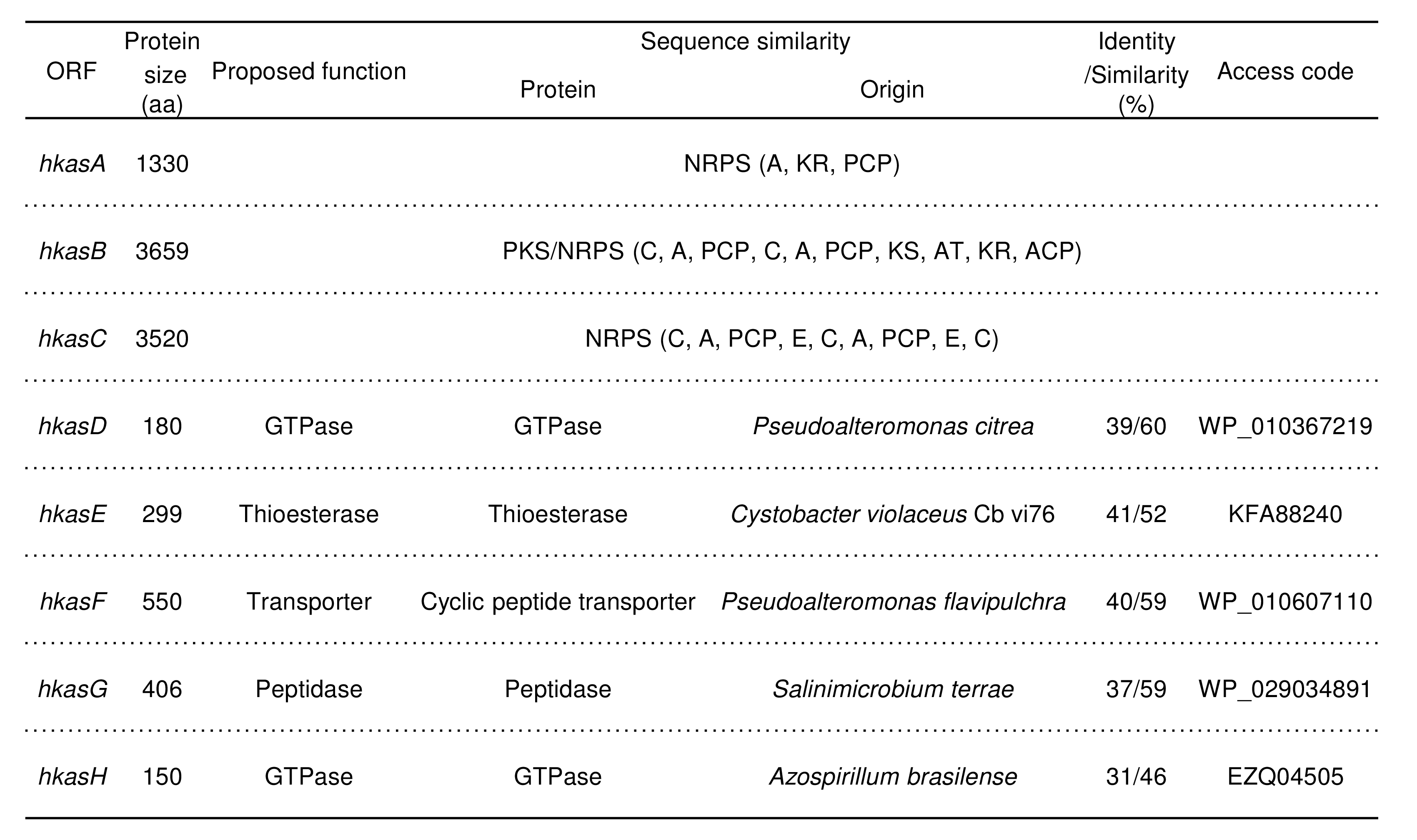

Supplement: S7 Table — CF444. (TIFF) [file pone.0164468.s021.tiff]
